# Neuropeptide Y‐Mediated Gut Microbiota Alterations Aggravate Postmenopausal Osteoporosis

**DOI:** 10.1002/advs.202303015

**Published:** 2023-10-19

**Authors:** Zhijie Chen, Mengyuan Lv, Jing Liang, Kai Yang, Fan Li, Zhi Zhou, Minglong Qiu, Haoyi Chen, Zhengwei Cai, Wenguo Cui, Zhanchun Li

**Affiliations:** ^1^ Department of Orthopaedic Surgery Renji Hospital School of Medicine, Shanghai Jiao Tong University 200127 Shanghai P. R. China; ^2^ Department of Orthopaedics Shanghai Key Laboratory for Prevention and Treatment of Bone and Joint Diseases Shanghai Institute of Traumatology and Orthopaedics Ruijin Hospital Shanghai Jiao Tong University School of Medicine 197 Ruijin 2nd Road Shanghai 200025 P. R. China

**Keywords:** brain‐gut‐bone axis, gut microbiota, neuropeptide Y, osteoblast pyroptosis, postmenopausal osteoporosis

## Abstract

Postmenopausal osteoporosis (PMO) is often accompanied by neuroendocrine changes in the hypothalamus, which closely associates with the microbial diversity, community composition, and intestinal metabolites of gut microbiota (GM). With the emerging role of GM in bone metabolism, a potential neuroendocrine signal neuropeptide Y (NPY) mediated brain‐gut‐bone axis has come to light. Herein, it is reported that exogenous overexpression of NPY reduced bone formation, damaged bone microstructure, and up‐regulated the expressions of pyroptosis‐related proteins in subchondral cancellous bone in ovariectomized (OVX) rats, but Y1 receptor antagonist (Y1Ra) reversed these changes. In addition, it is found that exogenous overexpression of NPY aggravated colonic inflammation, impaired intestinal barrier integrity, enhanced intestinal permeability, and increased serum lipopolysaccharide (LPS) in OVX rats, and Y1Ra also reversed these changes. Most importantly, NPY and Y1Ra modulated the microbial diversity and changed the community composition of GM in OVX rats, and thereby affecting the metabolites of GM (e.g., LPS) entering the blood circulation. Moreover, fecal microbiota transplantation further testified the effect of NPY‐mediated GM changes on bone. In vitro, LPS induced pyroptosis, reduced viability, and inhibited differentiation of osteoblasts. The study demonstrated the existence of NPY‐mediated brain‐gut‐bone axis and it might be a novel emerging target to treat PMO.

## Graphical abstract

A NPY‐mediated brain‐gut‐bone axis has come to light, demonstrating that the overexpression of NPY released by the hypothalamus induces intestinal inflammation and microbiota dysbiosis, where the “leaky gut” releases LPS to induce osteoblast pyroptosis and aggravate osteoporosis in OVX rats. It highlights the regulation of NPY neuroendocrine system in gut microbiota may be a novel emerging target to treat postmenopausal osteoporosis.

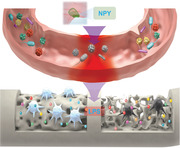

## 1 Introduction

Postmenopausal osteoporosis (PMO), mainly stems from the termination of ovarian function, where long‐term systemic low‐grade inflammation persists and results in stimulated bone resorption and reduced bone formation, leading to the deteriorated bone microstructure and bone loss.^[^ ^1^ ^]^ As is widely known for its severe bone quality impairment and bone fragility, PMO commonly comes with the decreased autonomy, chronic pain, susceptibility to fractures, exorbitant healthcare costs, and even high mortality.^[^ ^2^ ^]^ There is a bidirectional bone‐brain axis between the bone (known as a potential new endocrine organ) and the central nervous system.^[^ ^3^ ^]^ PMO patients are usually accompanied by neuropsychiatric disorders, including anxiety, depression, stress, and even neurodegenerative disorders,^[^ ^4^, ^5^, ^6^ ^]^ which can cause neuroendocrine changes in the hypothalamus,^[^ ^7^ ^]^ resulting in changes in hormones and peptide substances in the blood circulation, such as neuropeptides. Neuropeptides include neuropeptide Y (NPY), vasoactive intestinal peptide, calcitonin gene‐related peptide and substance P. Studies have shown that there are both positive and negative feedback from their regulation on the changes of bone mass in PMO patients.^[^ ^8^ ^]^ Likewise, our previous study has showed that neuropeptides in ovariectomized (OVX) rats are related to bone microstructure and pain threshold.^[^ ^9^ ^]^ Among them, NPY is closely associated with chronic pain in the musculoskeletal system,^[^ ^10^ ^]^ and it has key interactions with the sex steroid pathway in the homeostasis of bone and adipose tissue.^[^ ^11^, ^12^ ^]^ However, its effects on bone metabolism have not been fully understood.

NPY is a multi‐functional neuropeptide that is abundant in brain, gut, and bone tissue. It signals through five G‐protein coupled receptors (Y1, Y2, Y4, Y5, and Y6) and takes part in various physiological or pathological processes, regulating intestinal inflammation, bone metabolism, pain perception, brain function, and behavior.^[^ ^13^, ^14^, ^15^ ^]^ The hypothalamus secretes NPY and simultaneously regulates bone mass in response to changes of NPY levels in the circulation, which supports the existence of the interaction between neuroendocrine signals and bone metabolism.^[^ ^16^ ^]^ The expression of NPY, which will increase with aging and osteoporosis, together with its receptor Y1R, plays a strong part in bone metabolism. The overexpression of NPY in the hypothalamic arcuate nucleus in mice manifested antianabolic effects in bone with decreased osteoblast activity and reduced bone mass.^[^ ^17^ ^]^ Nicola et al.^[^ ^18^ ^]^ took virus‐mediated Y receptor‐knockout mice to simulate hypothalamic NPY overexpression in obese mice, and found that Y2/Y4 receptor double‐knockout mice exaggerated to the anti‐osteogenic effects (both cancellous and cortical bone) caused by elevated hypothalamic NPY, which appears to depend on Y1R signaling. However, researchers only focused on the exploration of direct effect of NPY on bone remodeling, but ignored the indirect effect of NPY‐mediated changes in gut microbiota (GM) on bone metabolism. The brain and GM interact through a variety of bidirectional signaling pathways, where NPY plays a potentially important role as a mediator.^[^ ^19^, ^20^ ^]^ Furthermore, increasing researchers have tried to elaborate the potential mechanisms of effects of GM on PMO based on the influence of brain‐gut‐bone axis on bone metabolism,^[^ ^21^, ^22^, ^23^, ^24^ ^]^ including the aspects of regulation of intestinal metabolites, regulation of intestinal epithelial barrier functions, neuroregulation, immune regulation, and endocrine regulation, etc. With the emerging role of the GM, a potential NPY‐mediated brain‐gut‐bone axis has come to light.

Recently, accumulating evidence has manifested that there is an inextricable crosstalk between the GM and PMO. Even a tiny perturbation of the GM can make for what initiates and reinforces the disruption of the bone homeostasis during the development of PMO.^[^ ^25^ ^]^ Therefore, deciphering the mechanism how GM controls bone remodeling fate switching is expected to develop GM‐based therapies against PMO. The GM refers to many species of microorganisms including bacteria, fungi, and viruses,^[^ ^26^ ^]^ regulating multiple biological pathways,^[^ ^27^ ^]^ such as microbial metabolites circulation, intestinal permeability, inflammatory and immune responses, and endocrine function, etc. There is increasing evidence that GM regulates bone metabolism via the gut‐bone axis, which is a promising target to treat PMO.^[^ ^28^ ^]^ For example, Li et al.^[^ ^29^ ^]^ demonstrated that sex steroid deficiency in germ‐free mice failed to trigger inflammatory pathways which are critical in PMO, highlighting the role that the GM and increased gut permeability play in estrogen deficiency–associated bone loss. Moreover, GM‐based therapies, such as probiotic *Lactobacillus reuteri*, yeast probiotic *Saccharomyces boulardii* and probiotic *Lactobacillus rhamnosus GG* can decrease gut permeability, dampen intestinal and bone marrow inflammation, reduce inflammatory osteoclastogenesis and protect from bone loss in estrogen‐deficient female mice.^[^ ^29^, ^30^, ^31^ ^]^ Although the critical role of GM on bone metabolism has been adumbrated by these pioneering researches, they mainly concentrated on the effect of intestinal metabolites on osteoclastic lineage, ignoring the irreplaceable role of osteoblastic lineage on the balance of bone remodeling in PMO.^[^ ^29^, ^32^, ^33^ ^]^ These findings motivated us to investigate whether osteoblasts are capable of functioning through gut‐bone axis to affect osteoporosis fate and bone formation‐resorption balance in PMO.

**Scheme 1:**
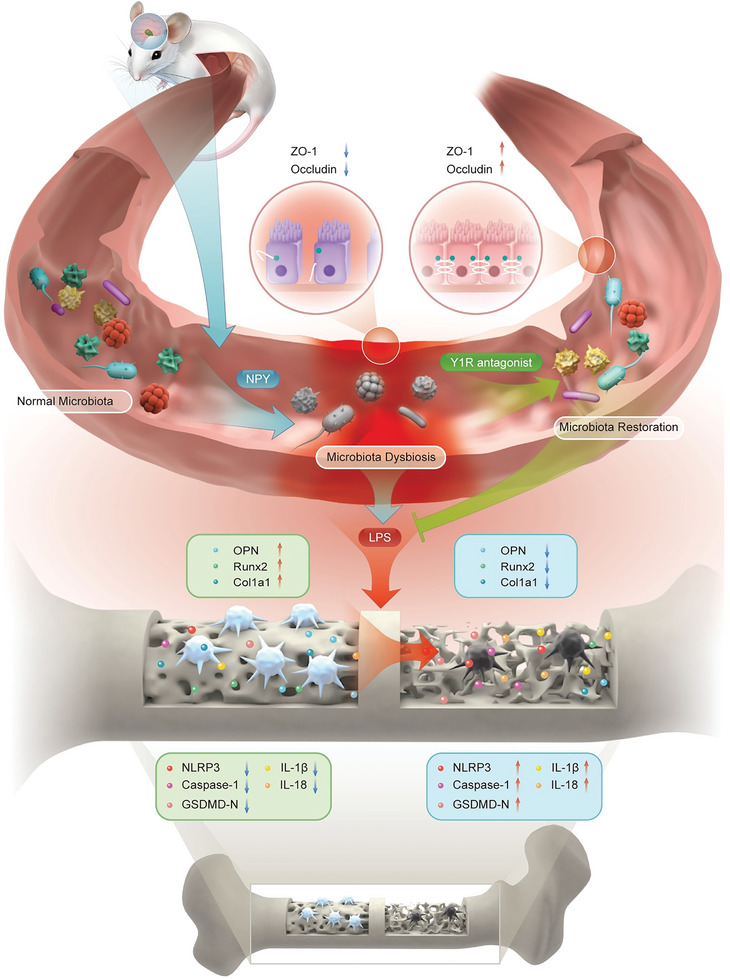
An overexpression of NPY modulates the microbial diversity and community composition of gut microbiota (GM) in OVX rats, which leads to microbiota dysbiosis, aggravates colonic inflammation, enhances intestinal permeability and thereby facilitates the metabolites of GM entering the blood circulation, such as lipopolysaccharide (LPS), etc. LPS induces pyroptosis, reduces viability and inhibits differentiation of osteoblasts, which aggravates osteoporosis in OVX rats. However, Y1 receptor antagonist (Y1Ra) reversed these changes.

Herein, we report that exogenous overexpression of NPY decreased bone formation and deteriorated bone microstructure while Y1 recptor antagonist (Y1Ra, BIBO3304) reduced bone loss and improved bone microstructure in OVX rats (**Scheme** **1**). We identify that the bone microstructure and pyroptosis‐related proteins expression in subchondral cancellous bone were modulated by NPY and Y1Ra. Furthermore, we demonstrate that exogenous overexpression of NPY aggravated colonic inflammation, damaged the integrity of intestinal barrier, and increased serum lipopolysaccharide (LPS), interleukin‐1β (IL‐1β), and interleukin‐18 (IL‐18), while Y1Ra reversed these changes in OVX rats. NPY and Y1Ra could modulate the microbial diversity and change the community composition of GM in OVX rats. Specifically, NPY increased *Firmicutes*/*Bacteroidetes* ratio, upregulated maleficent bacteria (e.g., *Desulfovibrionaceae*, producing LPS) and withhold probiotics (e.g., *Lactobacillus*, producing short‐chain fatty acids), while Y1Ra reversed these trends in OVX rats. Fecal microbiota transplantation from OVX rats differentially treated by exogenous overexpression of NPY or Y1Ra testified the impacts of GM on bone microstructure and bone mass. In vitro, the LPS was demonstrated to induce osteoblast pyroptosis, reduce cell viability and inhibit osteoblastic differentiation. Our study provides evidence that the intervention in NPY‐mediated brain‐gut‐bone axis may be a prospective strategy to treat PMO, and highlights the regulation of NPY/Y1Ra neuroendocrine system in GM as a new promising target.

## 2 Results and Discussion

### 2.1 NPY Decreased Bone Formation while Y1Ra Reduced Bone Loss in OVX Rats

**Figure 1:**
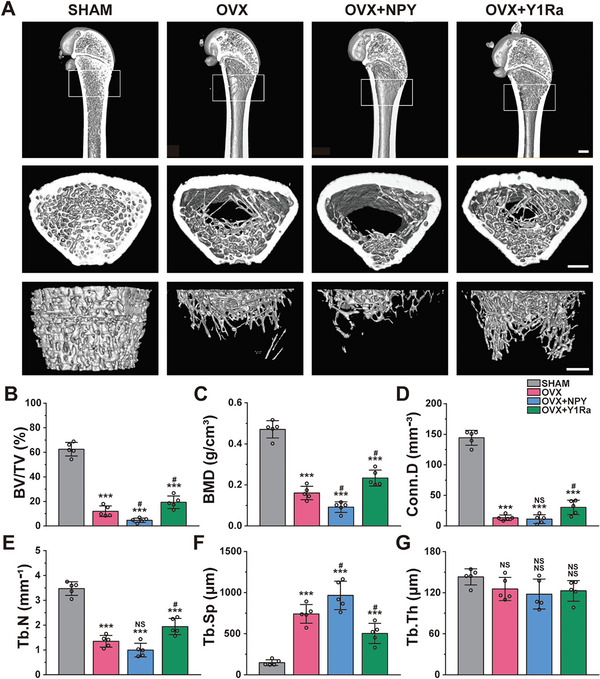
NPY decreased bone formation and deteriorated bone microstructure while Y1Ra was able to improve bone microstructure and reduce bone loss in OVX rats (*n* = 5). A) Representative Micro‐CT images of the structures of proximal tibia trabecular bone (scale bar = 1 mm); B–G) Quantitative analysis of bone mass and microstructures of rats influenced by OVX, NPY or Y1Ra; ^#^ *p* < 0.05 versus OVX.

In order to simulate estrogen deficiency caused PMO in this study, the rats were ovariectomized (OVX). The Micro‐CT imaging was used to analyze the right tibias subchondral cancellous bone of rats, which revealed detailed information about the bone mass and bone microstructure (**Figure** **1**A). Both 3D and 2D images were obtained and the amount and morphology of bone trabecula were quantitatively analyzed based on micro‐architectural parameters. Compared with the sham‐operation rats (the SHAM group), the OVX rats displayed significant reductions of bone mineral density (BMD), bone volume fraction (BV/TV), connectivity density (Conn.D), bone trabecular number (Tb. N), and bone trabecular thickness (Tb. Th), which presented osteoporotic changes of both bone mass and bone microstructure (Figure 1B–G). Furthermore, the indices were even lower in the OVX+NPY rats (Table S1, Supporting Information). However, the bone morphology was improved and the mass of bone trabecula increased in the OVX+Y1Ra rats.

### 2.2 The Bone Microstructure and Pyroptosis‐Related Proteins Expression in OVX Rats

**Figure 2:**
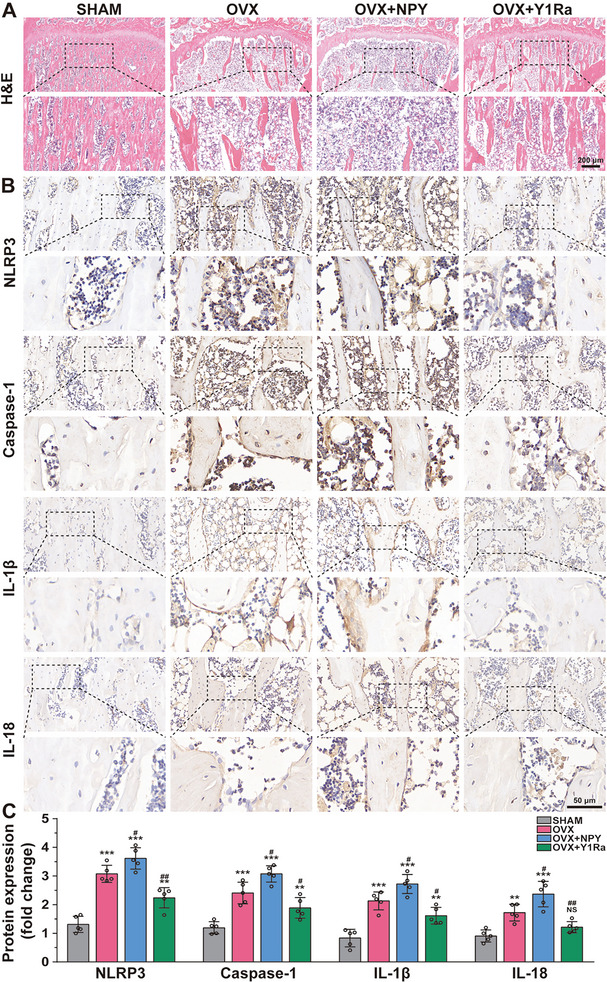
The bone microstructure and pyroptosis‐related protein expression in subchondral cancellous bone were modulated by NPY and Y1Ra in OVX rats (*n* = 5). A) Representative images and magnification presentations showing H&E staining of proximal tibia trabecular bone; B) Representative immunohistochemical images and magnification presentations showing the pyroptosis‐related protein expression in subchondral cancellous bone; C) The quantitative analysis showing the relative pyroptosis‐related proteins expression. **p < 0.01,***p < 0.001 versus SHAM; ^#^ *p* < 0.05, ^##^ *p* < 0.01,^###^ *p* < 0.001 versus OVX.

With the aim of studying deeply the effects of NPY and Y1Ra on bone mass and microstructure, we performed the histological assessment using H&E staining (**Figure** **2**A), as well as Masson's trichrome staining (Figure S1, Supporting Information). The results showed that regular mesh structure lost with sparsely and irregularly arranged trabecular bone in OVX rats. Almost no cancellous bone was found in the bone marrow spaces of OVX+NPY rats, which indicated that NPY aggravated osteoporosis in OVX rats. However, the microstructure of bone trabeculae was improved and the bone loss was suppressed in the OVX+ Y1Ra rats. All these results indicated that NPY and Y1Ra could change the bone microstructure.

As the long‐term systemic low‐grade inflammation persist in the PMO,^[^ ^1^ ^]^ we further examined the inflammasome activation in OVX rats. NOD‐like receptor protein 3 (NLRP3) inflammasome‐mediated pyroptosis is a recently discovered programmed process of cell death. However, it is not yet explicitly stated that there is pyroptosis in osteoblasts, which contribute to PMO.^[^ ^34^ ^]^ Exogenous damage like LPS and endogenous stimuli signals like ATP can induce common pathways to activate the NLRP3 inflammasome. NLRP3 promotes the cleavage of pro‐Caspase‐1 into biologically active Caspase‐1, which then cleaves gasdermin D (GSDMD, pore‐forming preprotein) to anchor and form pores on the cell membrane and concurrently leads to the cleavage of precursors IL‐1β and IL‐18 into mature ILs and their release through pores.^[^ ^35^, ^36^ ^]^ During this process, the balance of bone remodeling is disturbed with triggered osteoblasts death and amplified osteoclastic responses, which inhibits bone formation and promotes bone resorption. Studies showed that although direct bacterial infection was not observed in PMO, NLRP3 levels in osteoblasts still rose, with significantly decreased osteoblasts viability, and osteoporosis was also alleviated when NLRP3 was inhibited or inactivated.^[^ ^37^, ^38^, ^39^ ^]^ The underlying mechanism is still waiting in‐depth researches. In this study, the immunohistological analysis of NLRP3, Caspase‐1, GSDMD‐N and IL‐1β revealed an increase of pyroptosis in osteoblasts in OVX rats relative to the SHAM group (Figure 2B). As is shown, osteoblasts attached to the surface of bone trabeculae expressed increased proteins expression of genes related to pyroptosis. And, NPY aggravated the pyroptosis of osteoblasts in OVX rats. However, the expression of these proteins decreased in the OVX+Y1RA groups, which indicated that Y1Ra could mitigate the pyroptosis of osteoblasts in OVX rats. The quantitative analysis of relative proteins expression was exhibited in Figure 2C. All the results indicated that NPY and Y1Ra could modulate pyroptosis‐related protein expression in osteoblasts attached to the surface of subchondral cancellous bone in OVX rats.

### 2.3 Modulated Intestinal Permeability and Serum LPS, IL‐1β, and IL‐18

**Figure 3:**
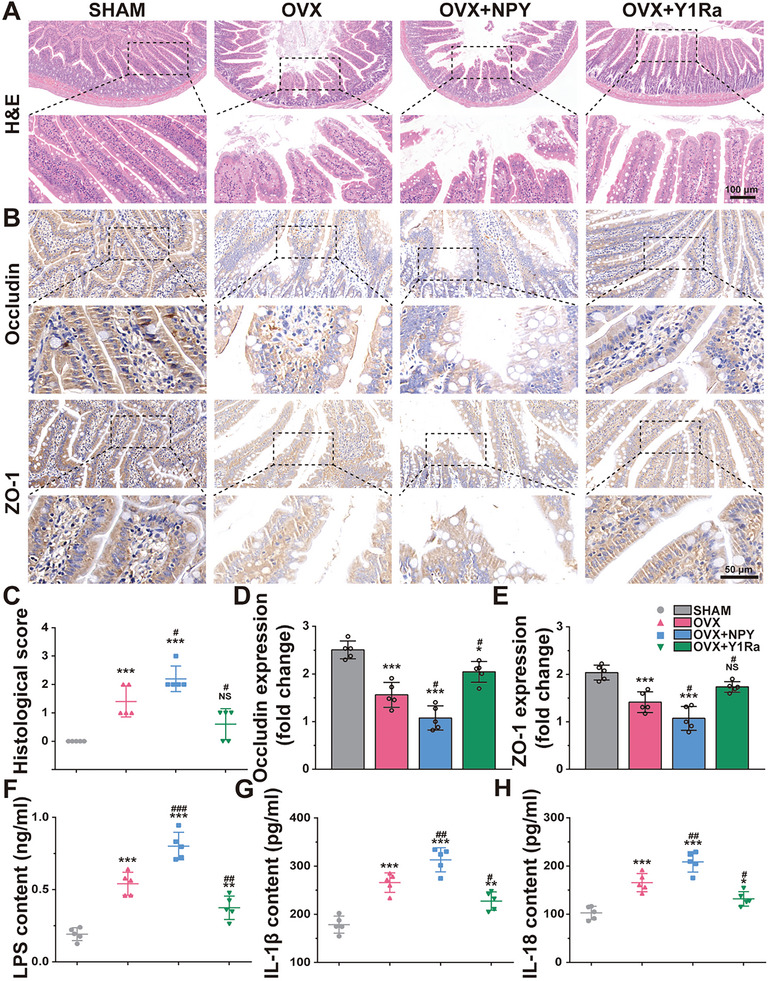
NPY and Y1R antagonist changed the intestinal barrier integrity in OVX rats (*n* = 5). A) Representative H&E histological images and magnification presentations of colons in rats; B) Representative immunohistochemical images and magnification presentations showing the TJ proteins (Occludin and ZO‐1) expression of colons in rats; C) Colonic histological scores based on H&E staining; D,E) The quantitative analysis showing the relative TJ protein expressions; F–H) The levels of LPS, IL‐1β, and IL‐18 in the serum detected by ELISA. *p < 0.05, **p < 0.01, ***p < 0.001 versus SHAM; ^#^ *p* < 0.05, ^##^ *p* < 0.01,^###^ *p* < 0.001 versus OVX.

Increasing evidence has suggested that estrogen deficiency can lead to GM imbalance, impaired intestinal barrier, and aggravated intestinal inflammation. In this process, intestinal metabolites from pathogenic intestinal bacteria can get released into the circulation via the damaged “leaky” intestinal barrier, further triggering the immune response, producing pro‐osteoclastogenic cytokines, damaging osteoblasts, and ultimately, leading to unbalanced bone remodeling and osteoporosis.^[^ ^29^, ^40^, ^41^ ^]^ This can highlight the existence of the gut‐bone axis and its unique significance. In our preceding review based on the gut‐bone axis, we summarized the regulatory role and significance of GM on osteoporosis, as well as the therapeutic potential of designing bioactive functional materials with living probiotics for osteoporosis.^[^ ^42^ ^]^ In this study, representative images of H&E‐staining and corresponding histopathological scores are shown in **Figure** **3**A,D. In the OVX group, the intestinal tissues manifested propria edema and mild inflammation, as reflected by epithelial cell injury and cellular mucin depletion. Furthermore, compared with the OVX group, the enteric cavities were sparser and the gaps of intestinal cells were more enlarged in the OVX+NPY group, which manifested an apparently compromised intestinal barrier. However, the impaired intestinal barrier integrity was improved in the OVX+Y1Ra group, as showed with increased intestinal villus height and decreased intestinal crypt depth, as well as higher intestinal villus density compared with the OVX group. Based on the colonic histopathological scores, the morphology damage of colonic tissues induced by ovariectomy was aggravated in the OVX+NPY group, while the intestinal injuries in OVX rats were repaired in the OVX+Y1Ra group.

Since the ovariectomy caused colonic changes led to the disruption of intestinal epithelial barriers in the view of histomorphology, the expression of tight junction (TJ) proteins between colonic epithelial cells in the colon, including Occludin and Zonula occludens‐1 (ZO‐1), were further analyzed to evaluate the intestinal barrier integrity. The immunohistological analysis displayed that compared with the SHAM rats, ovariectomy decreased the proteins expression of ZO‐1 and Occludin in the OVX rats (Figure 3B,C). In OVX+NPY rats, the protein expressions of Occludin and ZO‐1 were even lower compared with the OVX rats while they were reversed in the OVX+ Y1Ra rats, which demonstrated that the intestinal barrier function can be repaired by the Y1Ra via increasing the expression of intestinal adhesion protein in the OVX rats. In summary, OVX‐induced PMO jeopardized the intestinal barrier integrity and increased the intestinal permeability, NPY aggravated these changes while Y1Ra was able to restore them.

As it is well known, the metabolites of GM include LPS, short chain fatty acids, and bile acids, etc. Among them, LPS, also known as endotoxin, is the most clinically concerned and widely studied pathogenic substance, which can cause metabolic endotoxemia after entering the blood circulation.^[^ ^43^ ^]^ Studies have shown that LPS, a major component of the cell wall of Gram‐negative bacteria, can be recognized by intestinal epithelial cells and intestinal immune cells, stimulate inflammation by activating transforming growth factor and toll‐like receptor 4, thereby affecting host metabolism.^[^ ^44^ ^]^ The dysbiosis of GM can impair intestinal barrier integrity and enhance intestinal permeability, causing more LPS to enter the bloodstream.^[^ ^45^ ^]^ Therefore, in this study, we focused on LPS, which can cause cell death including apoptosis, programmed necrosis and pyroptosis, etc., thus aggravating tissue or organ damage.^[^ ^46^ ^]^ The inflammasome assembly in pyroptosis requires PAMPs, e.g., LPS, as the priming stimuli. During pyroptosis, pro‐inflammatory cytokines such as IL‐1β and IL‐18 were activated by inflammatory caspases and inflammasomes.^[^ ^47^ ^]^ As the intestinal permeability increased in the OVX rats, the contents of LPS, IL‐1β and IL‐18 in the serum were found to increase (Figure 3F–H). The results illustrated that the contents of LPS, IL‐1β, and IL‐18 in serum were higher in the OVX+NPY rats (0.80 ± 0.05 ng ml^−1^, 313.0 ± 11.2 pg ml^−1^, and 208.6 ± 9.5 pg ml^−1^) than those in OVX rats (0.54 ± 0.04 ng ml^−1^, 265.7 ± 8.9 pg ml^−1^, and 165.5 ± 8.5 pg ml^−1^), while they were reversed in the OVX+ Y1Ra rats (0.37 ± 0.04 ng ml^−1^, 227.4 ± 8.6 pg ml^−1^, and 131.9 ± 6.7 pg ml^−1^). In summary, NPY and Y1R antagonist were demonstrated to change intestinal homeostasis, intestinal barrier integrity, intestinal permeability and the levels of serum inflammatory cytokines derived from osteoblast pyroptosis in OVX rats.

### 2.4 Changed Community Compositions and Structures of GM

**Figure 4:**
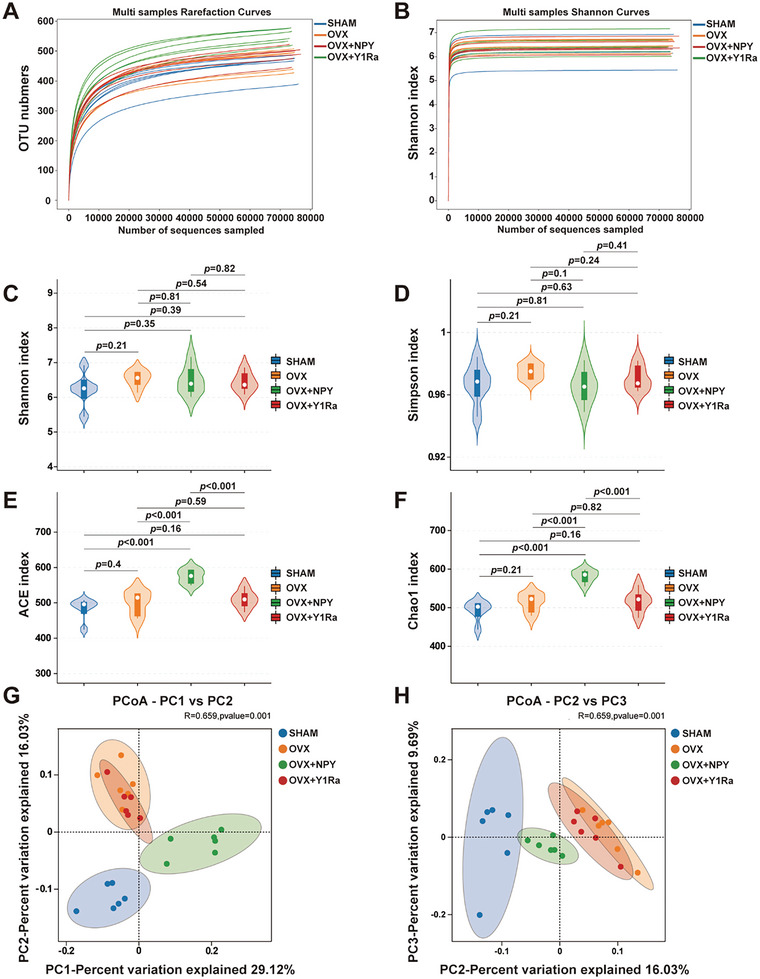
NPY and Y1R antagonist could modulate the microbial richness and diversity in OVX rats. The Rarefaction curves A) and the Shannon curves B) showed that the OTUs of the sample flora tended to be stable and the curves tended to be flat with the increase of sampling amount, indicating that the amount of sequencing data was sufficient, the sequencing depth of the sample was basically reached and the richness of species was adequate in the acquired samples; The Shannon index C) and the Simpson index D) showed no significant difference of the diversity of these groups, while the ACE index E) and the Chao1 index F) showed that the microbial species richness was significantly changed in OVX+NPY rats, which was reversed by Y1Ra, illustrating that NPY/Y1Ra could modulate the microbial species of OVX rats; The principal coordinate analysis (PCoA, PC1 vs PC2 and PC2 vs PC3, R = 0.659, *p* = 0.01) based on the Binary_Jaccard G,H) showed an apparent separation in the structure and the community composition of GM among four groups, illustrating that estrogen deficiency, NPY and Y1Ra were vital factors on the structures and the community compositions of GM in rats.

79861 clean reads every sample on average were obtained from 24 samples. As shown in **Figure** **4**A,B, the Rarefaction curves and Shannon curves reflected that the operational texonomic units (OTUs) of the sample flora tended to be stable and the curves tended to be flat with the increase of sampling amount, indicating that the amount of sequencing data was sufficient, the sequencing depth of the sample was basically reached and the richness of species was adequate in the acquired samples. The α‐diversity was estimated by Shannon index, Simpson index, ACE index and Chao1 index. The α‐diversity original data comparison among four groups were shown in Table S2 (Supporting Information). As illustrated in Figure 4C–F, the Shannon index and the Simpson index showed no significant difference of the diversity of these groups, while the ACE index and the Chao1 index showed that the microbial species richness was significantly changed in OVX+NPY rats, which was reversed by Y1Ra, illustrating that NPY/Y1Ra could modulate the microbial species of OVX rats. The principal coordinate analysis (PCoA) based on the algorithm of Binary_Jaccard distance was carried out to assess the community structures of GM (β‐diversity). The β‐diversity original data comparison among four groups were shown in Table S3 (Supporting Information). As shown in Figure 4G,H, the Binary_Jaccard metrics of PCoA (PC1 vs PC2 and PC2 vs PC3, R = 0.659, *p* = 0.01) showed apparent separation in the structure and the community composition of GM among four groups, which showed that estrogen deficiency, NPY and Y1Ra BIBO3304 were vital factors on the structures and the community compositions of GM.

**Figure 5:**
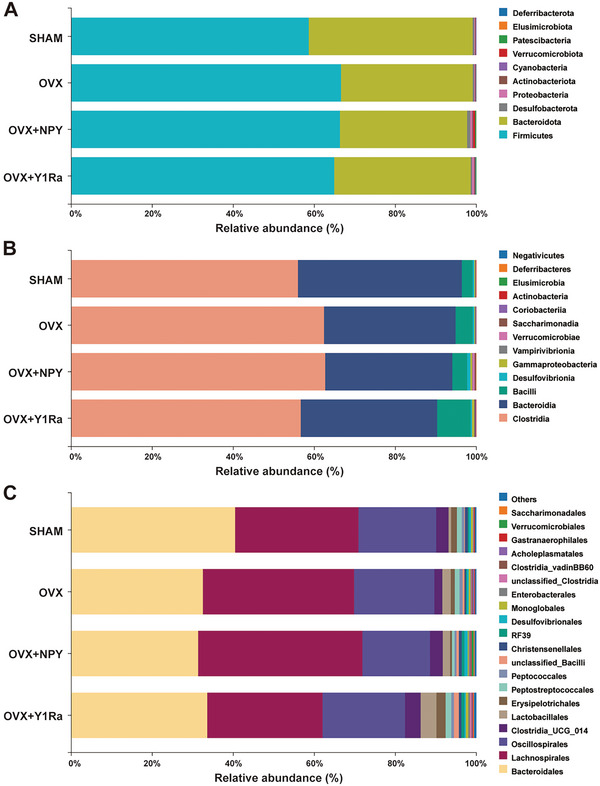
NPY and Y1R antagonist altered the microbial community composition and structure of GM in OVX rats (*n* = 6 per group). The composition and structure of GM at the phylum level A) showed that the ratios of *Firmicutes* to *Bacteroidetes* (F/B) in the OVX rats (2.05) and the OVX+NPY rats (2.11) were significantly higher than that in the SHAM rats (1.45), while it was reversed in the OVX+Y1Ra rats (1.92); The composition and structure of GM at the class level B) showed that the main proportions in SHAM rats (*Clostridia*: 56.0% and *Bacteroidia*: 40.5%) was significantly changed in the OVX rats (*Clostridia*: 62.4% and *Bacteroidia*: 32.5%) and the OVX+NPY rats (*Clostridia*: 62.6% and *Bacteroidia*: 31.4%),while they were reversed in the OVX+ Y1Ra rats (*Clostridia*: 56.6% and *Bacteroidia*: 33.7%); The composition and structure of GM at the order level C) showed that the main species abundance proportions in SHAM rats (*Bacteroidales*: 40.4%, *Lachnospirales*: 30.5% and *Oscillospirales*: 19.2%) was significantly changed in the OVX rats (*Bacteroidales*: 32.5%, *Lachnospirales*: 37.3% and *Oscillospirales*: 19.8%) and the OVX+NPY rats (*Bacteroidales*: 31.3%, *Lachnospirales*: 40.6% and *Oscillospirales*: 16.7%),while they were reversed in the OVX+ Y1Ra rats (*Bacteroidales*: 33.5%, *Lachnospirales*: 28.4% and *Oscillospirales*: 20.3%).

At the phylum, class and order levels, the microbial taxonomic compositions are showed in **Figure** **5**. *Bacteroidetes* and *Firmicutes* were two predominant phyla at the phylum level in the four groups (Figure 5A). As is well known, the ratio of *Firmicutes* to *Bacteriodetes* negatively correlated with bone volume.^[^ ^48^ ^]^ And the levels of *Clostridia* and *Lachnospiraceae* were also negatively correlated with the bone mass.^[^ ^48^ ^]^ The species abundance of *Firmicutes* was 58.6% in SHAM rats, 66.6% in OVX rats and 66.4% in OVX+NPY rats. The species abundance of *Bacteroidetes* was 40.5% in SHAM rats, 32.5% in OVX rats and 31.3% in OVX+NPY rats. The ratio of *Firmicutes* to *Bacteroidetes* (F/B) between the SHAM rats (1.45) and OVX rats (2.05) was changed. The ratio of F/B was even higher in OVX+NPY rats (2.11). Compared with the OVX group, after the administration of BIBO3304, the species abundance of *Firmicutes* (64.9%) in OVX+Y1Ra rats showed lower, and the species abundance of *Bacteroidetes* (33.7%) in OVX+ Y1Ra rats showed higher. The ratio of F/B was reversed in the OVX+Y1Ra rats (1.93) compared with OVX rats and OVX+NPY rats. Moreover, at the class level (Figure 5B), compared to the SHAM rats (*Clostridia*: 56.0% and *Bacteroidia*: 40.5%), *Bacteroidia* (32.5%) (phylum *Bacteroidetes*) was lower in OVX rats, *Clostridia* (62.4%) (phylum *Firmicutes*) showed higher in OVX rats. However, they were reversed in the OVX+ Y1Ra rats (*Clostridia*: 56.6% and *Bacteroidia*: 33.7%). Although the microbial taxonomic composition at the phyla or class level was similar in terms of predominant phyla between the OVX group and the OVX+NPY group, NPY was still found to change the compositions, especially at the order level. As was illustrated in Figure 5C, at the order level, the species abundance of *Bacteroidales* (phylum *Bacteroidetes*) in OVX rats (32.5%) showed lower than that in SHAM rats (40.4%) and lowest in OVX+NPY rats (31.3%). The main species abundance proportions in SHAM rats (*Bacteroidales*: 40.4%, *Lachnospirales*: 30.5%, and *Oscillospirales*: 19.2%) was significantly changed in the OVX rats (*Bacteroidales*: 32.5%, *Lachnospirales*: 37.3%, and *Oscillospirales*: 19.8%) and the OVX+NPY rats (*Bacteroidales*: 31.3%, *Lachnospirales*: 40.6%, and *Oscillospirales*: 16.7%),while they were reversed in the OVX+ Y1Ra rats (*Bacteroidales*: 33.5%, *Lachnospirales*: 28.4%, and *Oscillospirales*: 20.3%). The original data for composition and structure of GM at the phylum, class and order level among four groups were shown in Tables S4–S6 (Supporting Information). In addition, the abundance changes at the genus level were illustrated in Figure S2 (Supporting Information). Notably, *Lactobacillus* was only 0.3% in OVX rats. However, the abundance of *Lactobacillus* in OVX+Y1Ra rats increased to 2.1%. As the *Lactobacillus* belong to probiotics, which produce beneficial effects on health by regulating the balance of GM in the gut, it may partly explain why OVX+Y1Ra rats owned higher bone mass than OVX rats.

**Figure 6:**
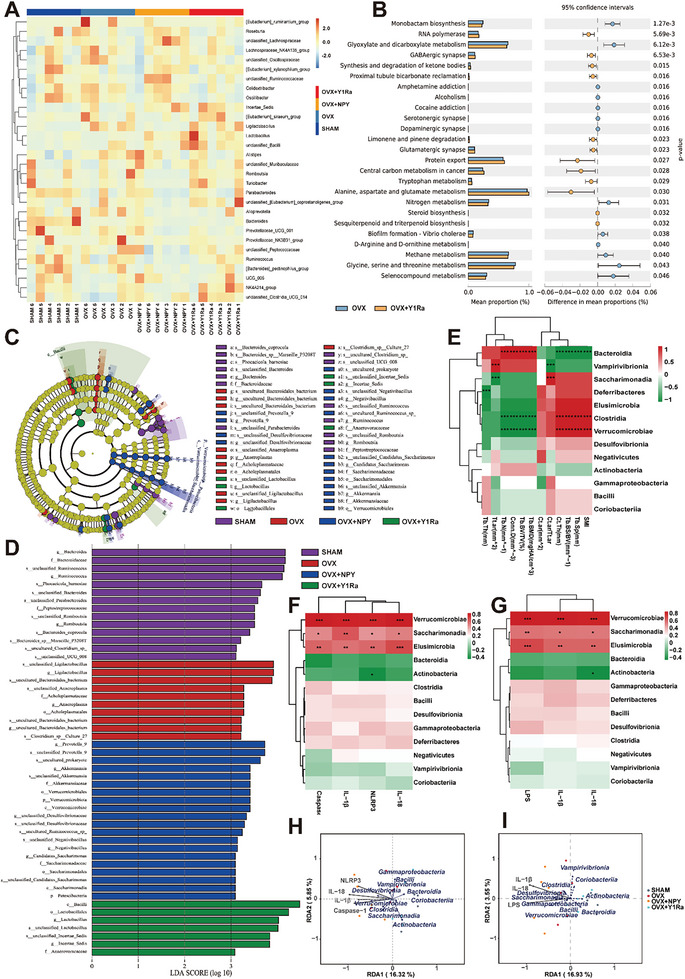
NPY and Y1Ra modulated genus‐level microbial communities and structure, changed predicted microbial metabolic functions and affected GM related to bone microstructure parameters. A) The 16S rDNA gene sequencing heat map of genus‐level GM (*n* = 6 of each group) showing 30 vital genera with significant difference in terms of the abundance (red colors mean high, whereas blue colors indicate low); B) The PICRUSt analysis result showing predicted microbial metabolic functional differences between the OVX+Y1Ra rats and the OVX rats based on KEGG database; C) The LEfSe analysis evolutionary branching diagram showing microbial communities with significant difference; D) The histogram of LDA value distribution of the LefSe analysis at a variety of taxonomic levels from phylum to species; E) The heat‐map of the correlation analysis of GM at the class level with bone mass and microstructure parameters (including Tb.BV/TV, Tb.BMD, Tb.Th, Tb.N, Tb.Sp, Conn.D, Tb.BS/BV, SMI, Ct.ar, Ct.Th, and Tt. Ar), illustrating that *Bacteroidia* positively correlated with the bone mass and microstructure, while both *Clostridia* and *Verrucomicrobiae* negatively correlated with bone mass and microstructure; F,G) The heat‐map of the correlation analysis of GM at the class level with pyroptosis related parameters (including Caspase‐1, NLRP3, IL‐1β, and IL‐18) and serum markers (including LPS, IL‐1β, and IL‐18), illustrating that *Verrucomicrobiae*, *Saccharimonadia* and *Elusimicrobia* positively correlated with them; H,I) The RDA analysis illustrating *Verrucomicrobiae*, *Saccharimonadia* and *Desulfovibrionia* closely correlated with pyroptosis related parameters (including Caspase‐1, NLRP3, IL‐1β, and IL‐18) and serum markers (including LPS, IL‐1β, and IL‐18).

To further identify the community composition and structure of GM altered in the OVX rats, a heat map was used to present the relative abundance of 30 genera (**Figure** **6**A). Compared to the OVX rats, the relative abundance of microbial compositional profiling of each sample changed by NPY and Y1R antagonist presented different community composition and structure of GM at the genus level. According to the Kyoto Encyclopedia of Genes and Gemos (KEGG) database, the Phylogenetic Investigation of Communities by Reconstruction of Unobserved states (PICRUSt) was performed to figure out the microbial functional differences and predict microbial metabolic functions among the four groups. The functional differences were compared between the OVX rats and the OVX+Y1Ra rats to explore whether the Y1Ra BIBO3304 in OVX rats modulated the microbial metabolic functions. It was predicted that compared with OVX rats, the function of aspartate, glutamate and alanine metabolism is significantly stronger and the function of serine, threonine and glycine metabolism is lower in OVX+Y1Ra rats (Figure 6B). In addition, compared with OVX rats, a variety of predicted functions changed a lot in OVX+Y1Ra rats, including RNA polymerase, GABAergic synapse, synthesis and degradation of ketone bodies, proximal tubule bicarbonate reclamation, limonene and pinene degradation, glutamatergic synapse, protein export, central carbon metabolism in cancer and tryptophan metabolism. Moreover, compared with OVX rats, the biosynthesis of secondary metabolism was predicted to be significantly stronger and the purine metabolism to be significantly lower in OVX+NPY rats (Figure S3, Supporting Information). The LDA Effect Size (LEfSe) analysis was further performed to analyze the results to figure out bacterial taxonomic markers related to OVX, NPY or Y1Ra. In order to discover the taxa differentially abundant at a variety of taxonomic levels from phylum to species, the histogram of linear discriminant analysis (LDA) value distribution of the LefSe analysis was performed to find out effect size in these groups (Figure 6C,D). The original data for LEfSe features of four groups were shown in Table S7 (Supporting Information). Specifically, compared with the OVX rats, *g_Lactobacillus*, *s_unclassified_Lactobacillus*, *o_RF39*, *f_uncultured_rumen_bacterium*, *g_uncultured_rumen_bacterium*, *s_uncultured_rumen_bacterium* and *f_Anaerovoracaceae* were found more enriched in the OVX+Y1Ra rats compared with the OVX rats (Figure S4A, Supporting Information). In addition, compared with the OVX rats, *c_Verrucomicrobiae*, *s_unclassified_Akkermansia*, *g_Akkermansia*, *o_Verrucomicrobiales*, *p_Verrucomivrobiota*, *f_Akkermansia*, *s_uncultured_Ruminococcus_sp*, *g_unclassified_Desulfovibrionaceae*, *s_unclassified_Desulfovibrionaceae*, *s_unclassified_Negativibacillus*, *g_Negativibacillus*, *g_unclassified_RF39*, *s_ unclassified_RF39*, *f_ unclassified_RF39* and *s_unclassified_prokaryote* were found more enriched in the OVX+NPY rats (Figure S4B, Supporting Information).

It was reported that LPS could be produced in quantity by the family *Desulfovibrionaceae* of GM, inducing potent inflammation in host bodies.^[^ ^49^ ^]^ Herein, compared with the OVX rats, a higher abundance of the family *Desulfovibrionaceae* was found in the OVX+NPY rats, while a lower abundance of the family *Desulfovibrionaceae* was found in the OVX+Y1Ra rats, contributing to less inflammation and reducing bone loss in OVX rats. Furthermore, the non‐metric multi‐dimensional scaling (NMDS) analysis (NMDS1 vs NMDS2, stress = 0.1256) based on the Binary_Jaccard showed an apparent separation in the structure and the community composition of GM between OVX rats and OVX+Y1Ra rats, illustrating that Y1Ra significantly changed the structures and the community compositions of GM in OVX rats (Figure S5A, Supporting Information). And the relative abundance of each sample within specific intestinal flora, which showed significant differences between the OVX and OVX+Y1Ra groups, is shown in Figure S6 (Supporting Information). On the other hand, the family *Lactobacilus* belonged to the probiotics, which benefited the bone formation and bone mineral density by regulating gut immune function, competitively inhibiting harmful microorganisms, and generating beneficial metabolites, such as short chain fatty acids (SCFAs).^[^ ^50^ ^]^ A higher abundance of the family *Lactobacilus* was found in the OVX+Y1Ra group (Figure 6C). The results showed that NPY and Y1Ra BIBO3304 in OVX rats could regulate microbial metabolic functions, and the Y1R antagonist reduced harmful bacteria that could produce harmful metabolites like LPS and promoted the growth of probiotics to competitively inhibit harmful bacteria, thus suppressing the progression of PMO.

Furthermore, the heat‐map of the correlation analysis of GM at the class level (Figure 6E) was performed with bone mass and microstructure parameters (including Tb.BV/TV, Tb.BMD, Tb.Th, Tb.N, Tb.Sp, Conn.D, Tb.BS/BV, SMI, Ct.ar, Ct.Th, and Tt. Ar). At the class level, *Bacteroidia* (phylum *Bacteroidetes*) exhibited significantly positively correlated with bone mass and microstructure related parameters (Tb.BV/TV, Tb.BMD, Tb.N, and Conn.D), while both *Clostridia* (phylum *Firmicutes*) and *Verrucomicrobiae* showed negative correlation with these parameters. The results further verified the conclusion that the ratio of *Firmicutes* to *Bacteriodetes* correlated negatively with bone mass. At the same time, the heat‐map of the correlation analysis of GM at the class level was investigated (Figure 6F,G) with pyroptosis related parameters (including Caspase‐1, NLRP3, IL‐1β and IL‐18) and serum markers (including LPS, IL‐1β, and IL‐18), illustrating that *Verrucomicrobiae*, *Saccharimonadia* and *Elusimicrobia* positively correlated with them. Accordingly, the redundancy analysis (RDA)/canonical correspondence analysis (CCA) was further investigated to explore the correlation (Figure 6H,I), which showed *Verrucomicrobiae*, *Saccharimonadia* and *Desulfovibrionia* closely correlated with pyroptosis related parameters (including Caspase‐1, NLRP3, IL‐1β and IL‐18) and serum markers (including LPS, IL‐1β and IL‐18). Notably, in this study, microbial enrichment analyses found out that c_*Verrucomicrobiae* (phylum *Acidobacteria*) significantly negatively correlated with bone mass and bone microstructure. Interesingly, not only *c_Verrucomicrobiae*, but also *o_Verrucomicrobiales*, *p_Verrucomivrobiota*, c_*Desulfovibrionaceae*, c_*Saccharimonadia, g_Desulfovibrionaceae*, *s_Desulfovibrionaceae* and c_*Desulfovibrionia* might be worthy of further study.

Collectively, these results illustrated that NPY and Y1Ra BIBO3304 could modulate the microbial diversity and changed the community composition of GM in OVX rats. In this way, they further regulated GM metabolites, intestinal permeability and intestinal homeostasis to affect bone metabolism through the gut‐bone axis. Thus, NPY can aggravate PMO, while Y1R antagonist can decrease inflammation and have an anti‐osteoporosis effect in OVX rats.

### 2.5 Fecal Microbiota Transplantation and Modulated Bone Mass and Microstructure

In order to further testify the effect of NPY‐mediated GM changes on the bone metabolism in the OVX mice, we performed fecal microbiota transplantation (FMT). Li et at.^[^ ^29^ ^]^ indicated that estrogen deficiency did not lead to the development of osteoporosis in germ‐free mice, and bone loss due to estrogen deficiency is mediated by GM. In their study, the independent variable is the change of GM (they also found that supplementation of exotic probiotics *Lactobacillus rhamnosus GG* in mice protects against bone loss). The present study differs from their study, and the independent variable of it is the change of NPY. Our receptors of FMT are all OVX rats. That means these rats all underwent ovariectomy. On the one hand, the depletion of GM might independently affect the bone mass in OVX rats. On the other hand, the depletion of GM might cause neuroendocrine changes (such as NPY) in the hypothalamus. These would interfere with our study, and the depletion of GM was abandoned. Therefore, the overall preparation of donor fecal transplant materials was referred to previous experimental researches with the similar assay.^[^ ^51^, ^52^, ^53^ ^]^

**Figure 7:**
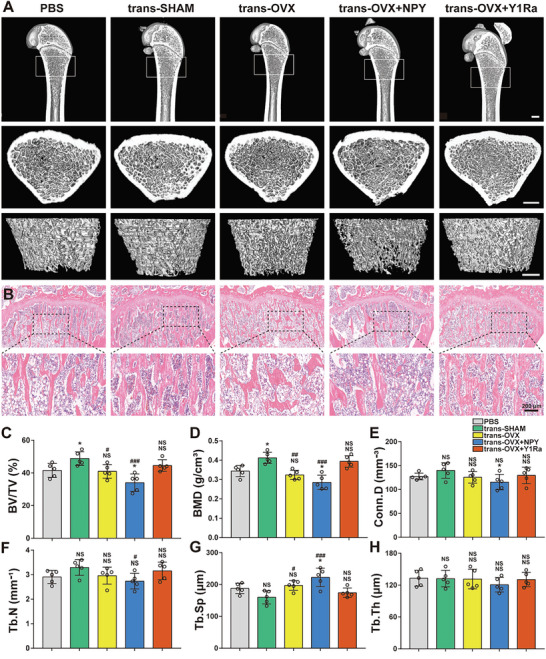
FMT from OVX rats influenced by PBS, NPY or Y1Ra modulated bone mass and bone microstructure during bone loss in OVX rats (*n* = 5). A) Representative images of Micro‐CT showing the structures of proximal tibia trabecular bone (scale bar = 1 mm); B) Representative images of H&E staining showing the structures of proximal tibia trabecular bone; C–H) Quantitative analysis of bone mass and microstructures of OVX rats influenced by FMT treated with PBS, NPY or Y1Ra. *p < 0.05 versus PBS; ^#^ *p* < 0.05, ^##^ *p* < 0.01, ^###^ *p* < 0.001 versus trans‐SHAM.

To be specific, the bacterial suspensions (mixed with PBS) from the feces of the groups of SHAM, OVX, OVX+NPY, and OVX+Y1Ra were transplanted to the recipient OVX rats (termed as trans‐SHAM, trans‐OVX, trans‐OVX+NPY, and trans‐OVX+Y1Ra) by gavage, and a group of OVX rats fed with PBS as the control was termed as the group of PBS. The Micro‐CT was performed for analysis of the subchondral cancellous bone of right tibias of these rats to detect the impacts of FMT on the bone mass and microstructure. Both three‐dimensional and two‐dimensional images were obtained (**Figure** **7**A) and the amount and morphology of bone trabecula were quantitatively analyzed based on micro‐architectural parameters. Compared to the rats in the PBS group, the tran‐SHAM rats exhibited decreased Tb.Sp and increased BV/TV, BMD, Conn.D, and Tb. N, which showed FMT from the normal rats ameliorated osteoporotic bone loss in OVX rats (Figure 7A). This was consistent with the study of Zhang et al,^[^ ^51^ ^]^ who found that FMT ameliorated bone loss in PMO mice. And the indices of the trans‐OVX rats were similar with rats in the PBS group. Of note, the rats in the trans‐OVX+NPY group exhibited least bone mass and worst bone microstructure, which showed that FMT from the OVX+NPY rats aggravated osteoporotic bone loss in OVX rats. However, these osteoporotic changes were greatly improved in the trans‐OVX+Y1Ra rats (Table S8, Supporting Information).

Furthermore, we performed H&E histological staining to further investigate the effect of NPY‐mediated GM changes on the bone mass or microstructure in the OVX rats (Figure 7B). The results showed that regular mesh structure lost with sparsely and irregularly arranged trabecular bone in rats of the PBS group and the trans‐OVX group. Less cancellous bone was found in the trans‐OVX+NPY rats, which indicated that FMT from the OVX+NPY rats aggravated osteoporotic bone loss in OVX rats. However, the bone loss was suppressed and the bone microstructure was improved in the bone trabeculae of trans‐OVX+Y1Ra rats. All these results indicated that NPY and Y1Ra could change the GM of OVX rats, which in turn affected the bone mass and bone microstructure.

Above studies have found that exogenous overexpression of NPY reduced bone formation, damaged bone microstructure and up‐regulated the expressions of pyroptosis‐related proteins in subchondral cancellous bone in OVX rats. At the same time, exogenous overexpression of NPY aggravated colonic inflammation, impaired intestinal barrier integrity, enhanced intestinal permeability, and increased serum LPS. However, whether LPS can cause osteoblast pyroptosis for aggravating osteoporosis in OVX rats and the corresponding specific molecular mechanisms have not been concluded in the international research field.^[^ ^34^ ^]^ The classical mechanism of LPS involvement in osteoblast pyroptosis may be as follows: LPS activates the NLRP3 inflammasome through the TLR4 receptor on the cell membrane, and produces biologically active Caspase‐1 for pyroptosis. However, apart from the classical pyroptosis signalling molecule Caspase‐1, Caspase‐11 may also mediate pyroptosis of osteoblasts through non‐classical pathways. The Caspase‐11 can directly recognize and bind to cytoplasmic LPS to initiate osteoblast pyroptosis by virtue of IFN‐γ.

In our study, preliminary researches were done to explore whether LPS would induce pyroptosis of osteoblasts. However, to explore in depth the molecular mechanisms between microbes and PMO, more researches await us. *c_Desulfovibrionaceae* is the focus of our initial consideration. This is because compared with OVX rats, a higher abundance of the *c_Desulfovibrionaceae* was found in the OVX+NPY rats, while a lower abundance of that was found in the OVX+Y1Ra rats. And Xiao et al.^[^ ^49^ ^]^ reported that *c_Desulfovibrionaceae* could produce LPS in quantity for inducing potent inflammation in host bodies. In subsequent experiments, we will construct corresponding gene knockout models (such as TLR4 gene knockout and Caspase‐11 gene knockout, etc.) at the cellular and animal levels to further verify the key molecular mechanisms. In the present study, serum LPS concentration was found to be as high as ≈1 ng mL^−1^ (Figure 3F) in the OVX + NPY rats. As a result, following this clue, the effects of 0, 0.1, 1,10 ng mL^−1^ LPS on the osteoblast fate were explored.

### 2.6 LPS Reduced Viability and Induced Pyroptosis of Osteoblasts

**Figure 8:**
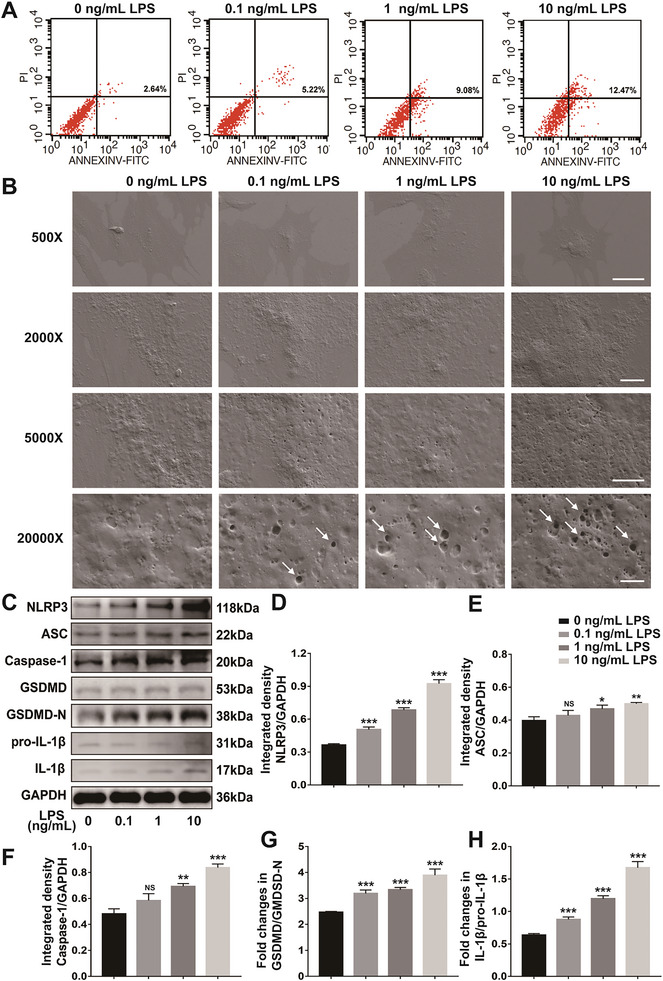
LPS reduced viability, induced pyroptosis and promoted the protein expressions of genes related to pyroptosis of osteoblasts. A) The results of flow cytometry showing the pyroptosis rate of osteoblasts treated with increasing concentration of LPS (the cell proportion in the right upper quadrant); B) Representative SEM results of osteoblasts under different LPS induction conditions [Scale bars = 50 µm (500×); Scale bars = 10 µm (2000×); Scale bars = 5 µm (5000×); Scale bars = 1 µm (20000×); the white arrows indicated pores on the cell membranes]; C) Representative western blot images to evaluate the protein expression levels of pyroptosis‐related genes including NLRP3, ASC, Caspase‐1, GSDMD, GSDMD‐N, pro‐IL‐1β, and IL‐1β in osteoblasts (GAPDH as the internal control); D–H) The quantitative analysis of relative proteins expression changes of ratios of NLRP3/GAPDH, ASC/GAPDH, Caspase‐1/GAPDH, GSDMD/GSDMD‐N, IL‐1β/pro‐IL‐1β in osteoblasts. *p < 0.05, **p < 0.01, ***p < 0.001.

The cell apoptosis was examined by flow cytometry after osteoblasts were intervened by LPS. As illustrated in **Figure** **8**A, the pyroptosis rate increased as the concentration of LPS increased from 0, 0.1, 1 to 10 ng mL^−1^. Meanwhile, the cell viability was measured by CCK‐8 test and Live/Dead assay. The cell viability of osteoblasts significantly decreased with treatment of LPS at the concentration from 0, 0.1, 1 to 10 ng mL^−1^ (Figure S7A, Supporting Information). The Live/Dead staining showed that many dead cells were observed but still quite a few seeded cells stayed alive over the course of 3‐day culture with 1 ng mL^−1^ LPS (Figure S7B, Supporting Information). In order to observe the phenomenon of pyroptosis more directly, the scanning electron microscope (SEM) was used to observe osteoblasts after LPS treatment. In the group of 0 ng mL^−1^ LPS as the control, the osteoblasts were in good condition. When the concentration of LPS was at 0.1 ng mL^−1^, osteoblasts begun to swell and form protrusions on the cell surface. Obviously, as the concentration of LPS increased, more and more pores formed on the membranes of osteoblasts, which would make the membranes of osteoblasts lose their integrities and the contents inside osteoblasts were released, subsequently causing pyroptosis. When the concentration of LPS reached 10 ng mL^−1^, the protrusions on the surface of osteoblasts and the swelling degree of osteoblasts also reached the peak, indicating that the pyroptosis was the most significant (Figure 8B). An essential marker protein NLRP3 during pyroptosis was studied to elucidate the effects of LPS on the proteins expression of pyroptosis in osteoblasts. The result of immunofluorescence staining showed that NLRP3 was visibly higher in the LPS group (1 ng mL^−1^) compared with the Control group (Figure S7C,D, Supporting Information). Furthermore, these pyroptosis related proteins produced by osteoblasts were semi‐quantitatively analyzed using western blot. The results illustrated that the expression levels of NLRP3, ASC, Caspase‐1, GSDMD‐N and IL‐1β of osteoblasts increased as the concentration of LPS increased from 0, 0.1, 1, to 10 ng mL^−1^ (Figure 8C–H). All the results indicated that LPS induced pyroptosis, reduced viability and promoted the protein expressions of genes related to pyroptosis of osteoblasts.

### 2.7 LPS Inhibited Osteogenesis and Suppressed Osteogenic Differentiation of Osteoblasts

**Figure 9:**
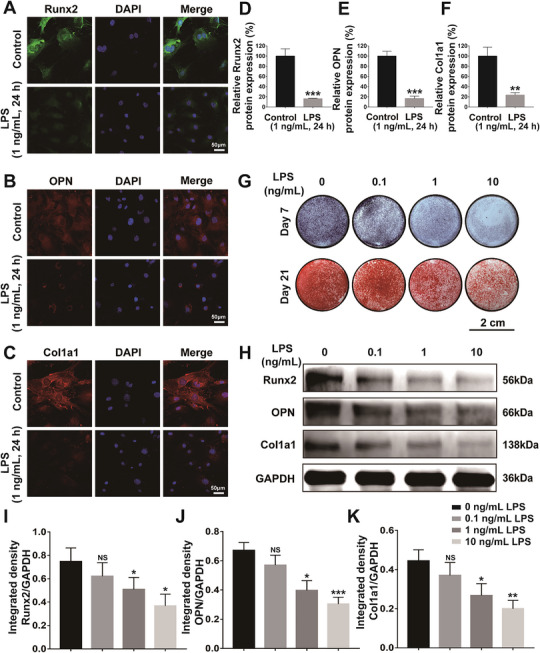
LPS inhibited osteogenesis and suppressed osteogenic differentiation of osteoblasts. A–C) Representative images of immunofluorescent staining showing the impacts of LPS on proteins expression of Runx2 (green), OPN (red), and Col1a1 (red) after 3‐day culture [cell nuclei were counterstained with DAPI (blue)]; D–F) The quantitative analysis showing the relative protein expression of Runx2, OPN and Col1a1 based on the fluorescence intensity; G) Representative digital images of ALP staining and Alizarin Red S staining showing the effects of LPS on the ALP activity and the production of extracellular mineralization matrix of osteoblasts after culturing for 7 days and 21 days; H) Representative western blot images to evaluate the proteins expression level of genes related to osteogenic differentiation, including Runx2, OPN and Col1a1 in osteoblasts (GAPDH as the internal control); I–K) The quantitative analysis of relative proteins expression changes of ratios of Runx2/GAPDH, OPN/GAPDH and Col1a1/GAPDH in osteoblasts. *p < 0.05, **p < 0.01, ***p < 0.001.

Several essential marker proteins during osteogenesis were studied to elucidate the impacts of LPS on the proteins expression of osteogenic differentiation in osteoblasts (**Figure** **9**). As demonstrated in Figure 9A–C, the results of immunofluorescence staining exhibited that all osteogenic differentiation related proteins expression of osteoblasts (cultured in osteo‐inductive media for 7 days), including osteopontin (OPN), runt‐related transcription factor 2 (Runx2), and Collagen type I (Col1a1), were visibly lower in the LPS group (1 ng mL^−1^) compared to the Control group. Correspondingly, the quantitative analysis of the fluorescence staining intensity showed that these proteins were significantly down‐regulated in the LPS group (Figure 9D–F). As an early osteogenesis‐related marker, alkaline phosphatase (ALP) has been widely recognized to reflect the stage of osteogenic differentiation. While endogenous bone mineralization derived from mineralized matrix production is also essential during osteogenesis, the Alizarin Red S staining was performed to detect the inorganic calcium to evaluate the efficiency of the mineralized nodules formation. With the concentration of LPS increasing (from 0 to 10 ng mL^−1^), ALP and mineralized nodules significantly decreased respectively after 7 days and 21 days (Figure 9G). Furthermore, these osteogenic differentiation related proteins produced by osteoblasts were semi‐quantitatively analyzed using western blot. The results illustrated that the expressed levels of Runx2, OPN and Col1a1 of osteoblasts decreased as the concentration of LPS increased from 0, 0.1, 1, to 10 ng mL^−1^ after culturing in osteo‐inductive media for 7 days (Figure 9H–K). All these results indicated that LPS inhibited osteogenesis and suppressed protein expressions of the osteogenic differentiation of osteoblasts.

## 3 Conclusion

Overall, we demonstrated a close relationship among neuroendocrine signals, GM and bone metabolism, providing evidence that the brain‐gut‐bone axis might serve as a promising target of the PMO treatment. Bone loss induced by estrogen deficiency in PMO might depend on GM, resulting in impaired intestinal barrier and increased circulating LPS. Exogenous overexpression of NPY aggravated colonic inflammation, damaged the integrity of intestinal barrier, and increased serum LPS, IL‐1β, and IL‐18, thus activating pyroptosis of osteoblasts, and further decreasing bone formation and deteriorating bone microstructure, while Y1R antagonist reversed these changes in OVX rats. These might be attributed to the modulation of the microbial diversity and the community composition of GM in OVX rats. Specifically, NPY increased *Firmicutes*/*Bacteroidetes* ratio, upregulated maleficent bacteria (e.g., *Desulfovibrionaceae*, producing LPS) and withhold probiotics (e.g., *Lactobacillus*, producing SCFAs), while Y1R antagonist reversed these trends in OVX rats. FMT from these rats further testified the impacts of GM on bone mass and microstructure. In vitro, the LPS was demonstrated to induce pyroptosis, reduce viability and inhibit differentiation of osteoblasts. Our study provides evidence that the intervention in NPY‐mediated brain‐gut‐bone axis may be a potential strategy for PMO treatment, and highlights the regulation of NPY neuroendocrine system in GM as a new promising target. In order to explore more conclusive clues, we have been planning to obtain bone tissue, stool, and blood samples from experimental animals, and to measure markers related to bone metabolism, including general biochemical markers, hormones regulating bone metabolism, markers of bone formation, and markers of bone resorption. At the same time, we plan to combine the sequencing analysis of multiple omics studies, including but not limited to metagenomics, 16S rDNA sequencing, metabolomics sequencing, transcriptomic sequencing, and proteomic sequencing, to further clarify the correlation between NPY‐related gut microbiota and PMO, and to elucidate which metabolic pathway is changed by NPY mediated brain‐gut‐bone axis. The validation of the findings in human cohort will be our ultimate pursuit.

## 4 Experimental Section

### Animal Groups

Institutional Animal Care and Use Committee of Shanghai Jiaotong University ethically approved of the animal experiments, which conformed to the National Guidelines for the Care and Use of Laboratory Animals in Research Institutions. All rats were maintained under standard laboratory conditions (humidity, 50 ± 5%; temperature, 25 ± 2 °C), and housed under the specific pathogen‐free (SPF) conditions, with a cycle of 12h:12 h light/dark. In addition, the cage effect should be taken into account, because the rodents in the same cage will share similar gut microbiota due to coprophagia, which can interfere in the study of microbiome in rodents.^[^ ^54^ ^]^ However, considering the cost and the space limitation of breeding site, single rat could not be completely raised in single cage. According to the experimental strategies of microbial researches summarized by predecessors,^[^ ^55^ ^]^ no more than two rats per cage will be kept in this study, to reduce the possible influence of cage effect on the experimental results. Twenty four 12‐week‐old virgin female SD rats were split into 4 groups in random (*n* = 6 per group): sham‐operation rats were defined as the SHAM group as control (adipose tissues of the same weight as the bilateral ovaries were resected); ovariectomized rats were defined as the OVX group (bilateral ovaries were excised); OVX rats administrated with Neuropeptide Y (Abcam, USA) (10 μg/kg/day, i.v.) were defined as the OVX+NPY group; OVX rats administrated with Y1R antagonist BIBO3304 (Tocris Bioscience, UK) (1 mg/kg/day, i.v.) were defined as the OVX+Y1Ra group. Drugs need to be administered to rats daily for 8 weeks after surgery for 4 weeks. After the administration of 8 weeks, fecal samples were harvested into sterile containers and frozen at − 80 °C immediately for 16S rDNA sequencing. During the 8‐week period of drug administration, fresh stool samples (from SHAM rats, OVX rats, OVX+NPY rats, and OVX+Y1Ra rats) were also daily collected and mixed with phosphate buffered saline (PBS) at a ratio of 10 mg/1 mL, vortexed and centrifuged to collect the supernatant to prepare stool suspensions for oral gavage. Before the FMT, these OVX rats were fed with the same food and water for at least 1 week. Therefore, it is assumed that the rats maintained similar baseline microbiota. The overall preparation of donor fecal transplant materials was based on previous experimental studies of similar trials.^[^ ^51^, ^52^, ^53^ ^]^ In general, twenty‐five 12‐week‐old virgin female SD rats (raised in the same environment) were split into 5 groups in random (*n* = 5 per group): the bilateral ovaries of these rats were first all excised, then they were administered with 100 µl (10^9^ CFU/mL) of stool suspensions by oral gavage every day for 8 weeks from rats of SHAM, OVX, OVX+NPY, and OVX+Y1Ra groups, which were defined as the groups of trans‐SHAM, trans‐OVX, trans‐OVX+NPY, and trans‐OVX+Y1Ra; the rats in the PBS group were administrated with 100 µl of PBS as control. To be more specific, the experiment cycle was at 9:00 a.m. every day, these OVX rats were induced to excrete fresh feces (10–20 pellets) by anal stimulation, then PBS was added, and the mixture was dissolved evenly (10 mg feces /1 mL PBS, vortex for ≈30 s, until there were no visible feces particles in the solution). Next, the obtained sample was centrifuged at 2000 rpm and 4 °C for 10 min, the fecal residue was discarded, and the supernatant was taken. The supernatant was then centrifuged again at 8000 rpm and 4 °C for 5 min to obtain the total bacterial count, subsequently mixed with PBS and prepared for further FMT treatment.

### Bone Micro‐CT Imaging

The right rat tibias were examined with the Micro‐CT system (Skyscan 1172, Aartselaar, Belgium) at 15‐µm voxel size. The region of interest (ROI) was chosen to analyze centered over the specimen after scanning. According to the ROI, the locational trabecular bone was reconstructed at 3D levels. The listed parameters were quantitatively analyzed by the software within the apparatus: BV/TV, BMD, Conn.D, Tb.Th, Tb.N, and Tb.Sp.

### Bone Histomorphometry

After decalcification, bone tissues of proximal left rat tibias were embedded with paraffin, sliced into 5‐mm sections, stained with H&E and Masson trichrome (Beyotime Biotech, Shanghai, China). Then, a digital microscope (Olympus, Japan) was used to observe the staining.

### Colonic Histological Assessment

The colon tissues were embedded with paraffin, sliced into 5‐mm sections, stained with H&E (Beyotime Biotech, Shanghai, China), and were evaluated by two observers individually in a blind manner. And the sections were assessed with rules of scoring, which are shown in detail in Table S9 (Supporting Information).

### Immunohistochemistry

According to the method reported previously, the immunohistochemistry of the proximal left tibias after decalcification and the colons of rats were performed and assessed.^[^ ^51^, ^56^, ^57^ ^]^ In brief, the sections were incubated with NLRP3 (1:200, Servicebio, China), Caspase‐1 (1:300, Servicebio, China), IL‐1β (1:100, Servicebio, China), IL‐18 (1:200, Servicebio, China), Occludin (1: 600, Servicebio, China), and ZO‐1 (1: 600, Servicebio, China), respectively. All images were captured by a LSCM (Zeiss, Germany).

### Assessment of Serum LPS, IL‐1β, and IL‐18 levels

Serum levels of LPS, IL‐1β, and IL‐18 were measured with ELISA from rats’ serum based on the manufacturer's instruction. The ELISA kits used were commercially available kits: rat LPS ELISA kits, rat IL‐1β ELISA kits, and rat IL‐18 ELISA kits (all from Cusabio, China).

### Analysis of Community Composition of GM by 16S rDNA Gene Sequencing

TGuide S96 Magnetic Soil/Stool DNA Kit (Tiangen Biotech, China) was used to extract the total microbial DNA from the fecal samples. According to standard protocols, an Illumina NovaSeq6000 platform at Biomarker Technologies was used to construct sequencing libraries and sequence paired ends. Paired‐end reads should be merged utilizing FLASH v 1.2.11, and tags exceeding six mismatches would be discarded. The tags merged shorter than 350 bps were deleted and those in a 50 bp sliding window with an average quality score <20 was determined with Trimmomatic. Operational taxonomic units (OTUs) with 97% similarity were clustered with the denoised sequences after the possible chimeras were removed performing on the USEARCH (version 10.0). Furthermore, the QIIME software was used to search against the Silva databases (Release138.1) for assigning the taxonomy to all OTUs. The α‐diversity analysis included the Rarefaction curves, the Shannon curves, the Shannon index, the Simpson index, the ACE index and the Chao1 index. The species richness and species diversity were characterized by these analyses. The EMPeror software was used to visualize the principal coordinate analysis (PCoA) plots based on the Binary_Jaccard (PC1 vs PC2 and PC2 vs PC3) for β‐diversity analysis. Through the analysis, the community structure component map, the species composition, and the community heatmap of GM could be obtained. Furthermore, based on the Kyoto Encyclopedia of Genes and Gemos (KEGG) database, PICRUSt was performed to predict microbial metabolic functions. Based on taxonomic composition in accordance with the different groups, LEfSe analysis and linear discriminant analysis (LDA) were performed on the samples to explore sample classification of the communities or species which showed significant differences. The correlations between GM and bone microstructure related parameters were investigated using correlation heat‐map analysis.

### Primary Calvarial Osteoblast Isolation, Culture and Stimulation

As described previously, primary calvarial osteoblasts isolation and culture were performed.^[^ ^58^, ^59^ ^]^ In brief, primary calvarial cells were separated from SD rats aged 1–3 days. Four sequential digestions were performed on parietal bones of calvariae after the sutures were removed. First, 1 mg mL^−1^ collagenase A and 2 mg mL^−1^ Dispase II (Roche, Indianapolis, USA) were mixed as an enzyme mixture to perform the first two digestions on a rocking platform at 37 °C. Second, 2 mg mL^−1^ collagenase A and 2 mg/mL Dispase II (Roche, Indianapolis, USA) were mixed as an enzyme mixture to perform the last two digestions. Then, a complete medium with an equal volume was used to stop enzyme activity after the osteoblasts fractions (3 to 4) were harvested. The medium contained α‐MEM (Gibco, USA), 10% FBS (HyClone, USA), and 1% penicillin/streptomycin (Gibco, USA). Then, these cell fractions were centrifuged, resuspended and filtered by a 70‐µm cell strainer using the complete medium. The harvested cells were cultured in the complete medium and differentiated with osteogenic medium. The medium contained 50 µm ascorbic acid, 10 mm β‐glycerophosphate and 10 nm dexamethasone (all from Sigma‐Aldrich, USA). 10^6^ cells per well (6‐well plates) or 10^5^ cells per well (24‐well plates) were seeded for all cell stimulation experiments. When grown to 80% confluence, cells were stimulated by 5 mm ATP for 30 min and then with 0, 0.1, 1, and 10 ng mL^−1^ LPS (Sigma‐Aldrich, USA). Then, cells were collected or fixed with paraformaldehyde for further experiments including cell viability test, flow cytometry, western blot, immunofluorescent staining, etc.

### Measurement of Cell Viability

As cultured to the confluence of 80%, the cells were stimulated by 5 mm ATP for 30 min and then with 0, 0.1,1, and 10 ng mL^−1^ LPS for 24 h. Then, the culture medium was cast away, and CCK‐8 reagent (ck04, Dojindo, Japan) was added and incubated in an incubator for 4 h at 37 °C. A microplate reader (TECAN, Switzerland) at 450 nm was utilized to quantify the cell viability. Additionally, to further assess osteoblasts viability, after stimulation by 5 mm ATP for 30 min and subsequently with 0 and 1 ng mL^−1^ LPS for 24 h, cells were dyed for 30 min employing a Live/Dead kit (Invitrogen, USA). The viable cells were stained by calcein AM (green) and dead cells were dyed by EthD‐1 (red) based on the manufacturer's protocol. A LSCM (Zeiss, Germany) was used to acquire the fluorescence images.

### Determination of Pyroptotic Cell Death

The flow cytometry was used to determine the ratio of pyroptotic cells.^[^ ^60^ ^]^ FITC and PI (Beyotime, Shanghai, China) were used to stain cells after they were digested by 0.025% trypsin. A flow cytometer (BD/FACS AriaIII, USA) was used to evaluate the cell pyroptosis rate, which was considered as cell proportions in the upper right quadrant.

### Scanning Electron Microscope (SEM) Observation

Osteoblasts were seeded onto the clean glass slides and stimulated by 5 mm ATP for 30 min and then with 0, 0.1, 1, and 10 ng mL^−1^ LPS for 24 h after they were cultured to the confluence of 60–70%. The cultured cells were rinsed by PBS, supplemented with pre‐cooled 3% glutaraldehyde, then fixed at 4 °C. Next, these cells were flushed by PBS, fixed with pre‐cooled 1% osmic acid at 4 °C. Then, they were dehydrated by a series of gradient alcohols. Subsequently, the critical point drying method was used to dry the samples. Finally, a SEM (SU8100, Hitachi, Japan) was used to observe the sample after they were coated with thin layers (5 nm) of platinum.

### Western Blot

The osteoblasts were treated by 5 mm ATP for 30 min and then with 0, 0.1, 1 and 10 ng mL^−1^ LPS for 24 h after they were grown to a confluence of 80%. Then, cell samples were lysed in RIPA buffer on ice, collected, and ultimately centrifuged for the removal of cell debris. A BCA Protein Assay Kit (Beyotime, China) was utilized for the determination of the protein concentrations, and predetermine and exclude the samples with low yield of proteins. SDS‐PAGE in a 10% gel and PVDF membranes (Millipore, USA) were successively used to separate and transfer each sample with 10 µg of total protein. TBST mixed by 5% dry nonfat milk was used to blockage the membranes. Then, the primary antibodies against Runx2 (1: 1000, Abcam, UK), Col1a1 (1: 1000, Abcam, UK), OPN (1: 1000, Abcam, UK), NLRP3(1: 200, Novus, USA), Caspase‐1 (1: 200, Santa Cruz, USA), ASC (1: 200, Santa Cruz, USA), GSDMD (1: 1000, Abcam, UK), IL‐1β (1: 1000, Abcam, UK), GAPDH (1: 1000, Abcam, UK) were used to incubate overnight the membranes at 4 °C. Next, the horseradish peroxidase–conjugated secondary antibodies (1: 4000, Santa Cruz, USA) were utilized to incubate the membranes at room temperature for 60 min after they were washed with TBST thrice. Finally, the antigen–antibody complexes were visualized by the enhanced chemiluminescence assay (Thermo Scientific, USA).

### Immunofluorescence Staining

After osteoblasts were cultured for 24 h, the complete medium was replaced by the osteogenic medium containing 50 µm ascorbic acid, 10 mm β‐glycerophosphate and 10 nm dexamethasone (all from Sigma‐Aldrich, USA). After being cultured for 7 days, osteoblasts were stimulated first by 5 mm ATP for 30 min and subsequently with 0 and 1 ng mL^−1^ LPS for 24 h. Afterward, osteoblasts were washed by PBS buffer trice, fixed with 4% paraformaldehyde, and then treated with 0.2% Triton X‐100. Next, a 10% goat serum solution (Invitrogen, USA) was used to block for nonspecific binding. The primary antibodies were then used to incubate overnight at 4 °C, including Runx2 (1:200, Abcam, UK), Col1a1 (1:200, Abcam, UK), OPN (1:200, Abcam, UK), and NLRP3 (1: 50, Novus, USA). The molecular probes Alexa Fluor‐coupled secondary antibodies (1:400, Life Tech, USA) were utilized to incubate the osteoblasts for 1 h at 37 °C after the incubation of primary antibodies. Subsequently, the DAPI was used to counterstain the Nuclei, and osteoblasts were washed with PBS thoroughly. Finally, a LSCM (Zeiss, Germany) was used for observation and qualitative analysis.

### Alizarin Red S (ARS) Staining and Alkaline Phosphatase (ALP) Staining

After osteoblasts were seeded, cultured and grown to a confluence of 60–70%, osteoblasts were stimulated by 5 mm ATP for 30 min and then the mediums were substituted by osteogenic mediums containing 0, 0.1, 1 and 10 ng mL^−1^ LPS. After being cultured for 7 days, an Alkaline Phosphatase Assay Kit (Beyotime, China) was utilized to examine the ALP activity. Then, after being cultured for 21 days, ARS staining (Cyagen Biosciences, China) was used to examine the mineralized nodule formation based on the manufacturer's protocol.

### Statistical Analysis

All data are present as mean ± standard deviation (SD) with three independent experiments. Two‐tailed Student's t‐test was used to determine significances between two groups. One‐way analysis of variance was utilized to perform the statistical comparisons between multiple groups. Detailed sample sizes were labeled in figures and the sample size for each statistical analysis (n) was at least three. GraphPad Prism 9.00 (USA) was utilized for data analysis. In term of all statistical tests, P value < 0.05 was considered as statistically significant.

## Conflict of Interest

The authors declare no conflict of interest.

## Supporting information

Supporting Information

## Data Availability

The data that support the findings of this study are available from the corresponding author upon reasonable request.
