# Supplementary material for: Neuropeptide Y‐Mediated Gut Microbiota Alterations Aggravate Postmenopausal Osteoporosis
Source: Adv Sci (Weinh). 2023 Oct 19;10(33):2303015. doi: 10.1002/advs.202303015 (PMC10667841; doi:10.1002/advs.202303015)
Supplement: Supplementary file 1 — Supporting Information [file ADVS-10-2303015-s001.pdf]

ADVANCED SCIENCE
Open Access

Supporting Information

for *Adv. Sci.*, DOI 10.1002/advs.202303015

Neuropeptide Y-Mediated Gut Microbiota Alterations Aggravate Postmenopausal Osteoporosis

Zhijie Chen, Mengyuan Lv, Jing Liang, Kai Yang, Fan Li, Zhi Zhou, Minglong Qiu, Haoyi Chen, Zhengwei Cai, Wenguo Cui* and Zhanchun Li**

Supplementary Information

Neuropeptide Y-mediated gut microbiota alterations aggravate postmenopausal osteoporosis

Zhijie Chen, Mengyuan Lv, Jing Liang, Kai Yang, Fan Li, Zhi Zhou, Minglong Qiu, Haoyi Chen, Zhengwei Cai, Wenguo Cui*, and Zhanchun Li**

Dr. Z. Chen, F. Li, Z. Zhou, and Prof. Z. Li
Department of Orthopaedic Surgery, Renji Hospital, School of Medicine, Shanghai Jiaotong University, Shanghai, 200127, China.
E-mail addresses: lzctgzyyx@163.com (Z. Li)

Dr. Z. Chen, Dr. M. Lv, Dr. J. Liang, Dr. K. Yang, Dr. M. Qiu, Dr. H. Chen, Dr. Z. Cai, and Prof. W. Cui
Department of Orthopaedics, Shanghai Key Laboratory for Prevention and Treatment of Bone and Joint Diseases, Shanghai Institute of Traumatology and Orthopaedics, Ruijin Hospital, Shanghai Jiao Tong University School of Medicine, 197 Ruijin 2nd Road, Shanghai, 200025, P. R. China.
E-mail addresses: caizhengwei@shsmu.edu.cn (Z. Cai), wgcui80@hotmail.com (W. Cui)

Keywords: Neuropeptide Y; Gut microbiota; Postmenopausal osteoporosis; Brain-gut-bone axis; Osteoblast pyroptosis

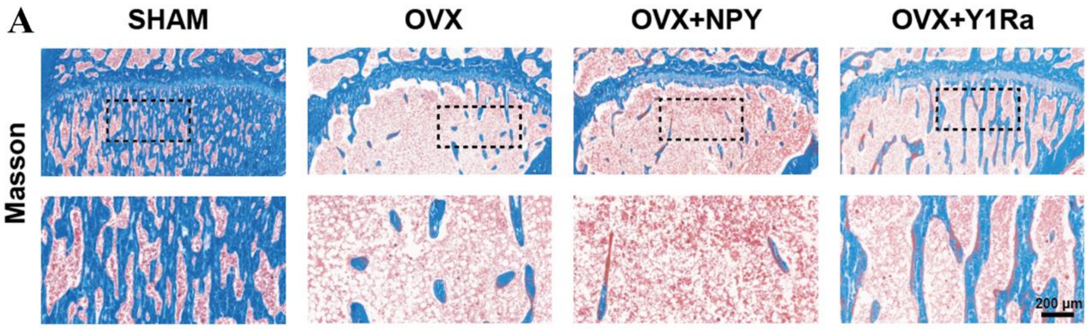

Supplementary Figure S1. Masson's staining of proximal tibia trabecular bone. **(A)** Representative images of the groups of SHAM, OVX, OVX+NPY and OVX+Y1Ra

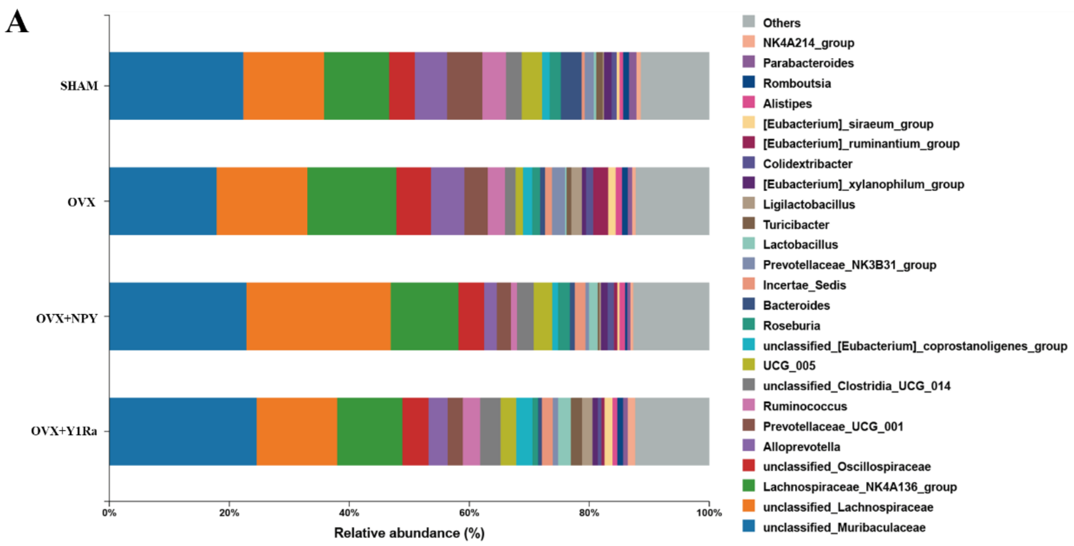

Supplementary Figure S2. The microbial taxonomic compositions at the genus level. **(A)** The composition of gut microbiota of the groups of SHAM, OVX, OVX+NPY and OVX+Y1Ra at the genus level.

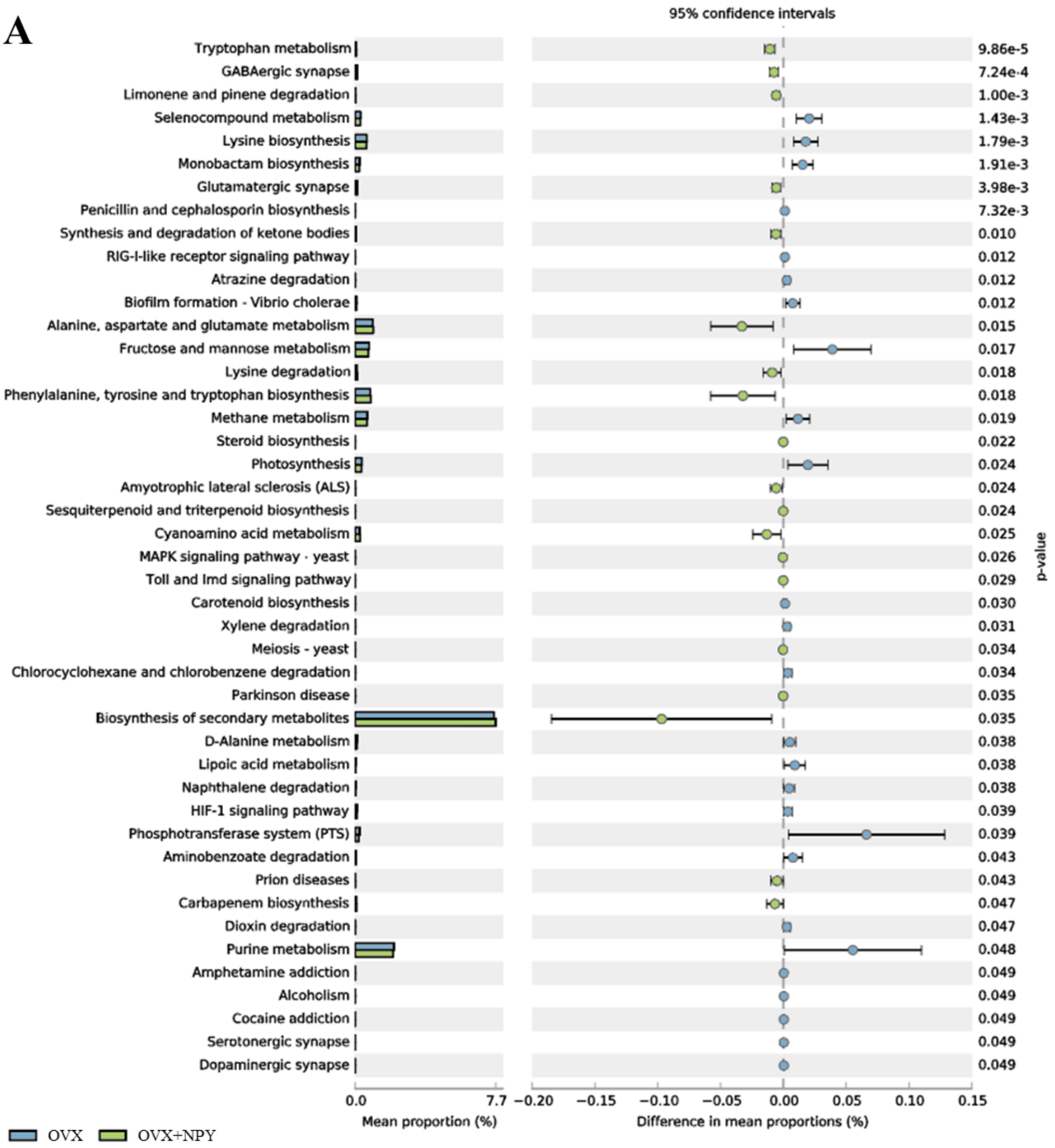

Supplementary Figure S3. The PICRUSt analysis based on KEGG database**. (A)** The PICRUSt analysis between the OVX+NPY group and the OVX group to predict microbial metabolic function and analyze the functional differences.

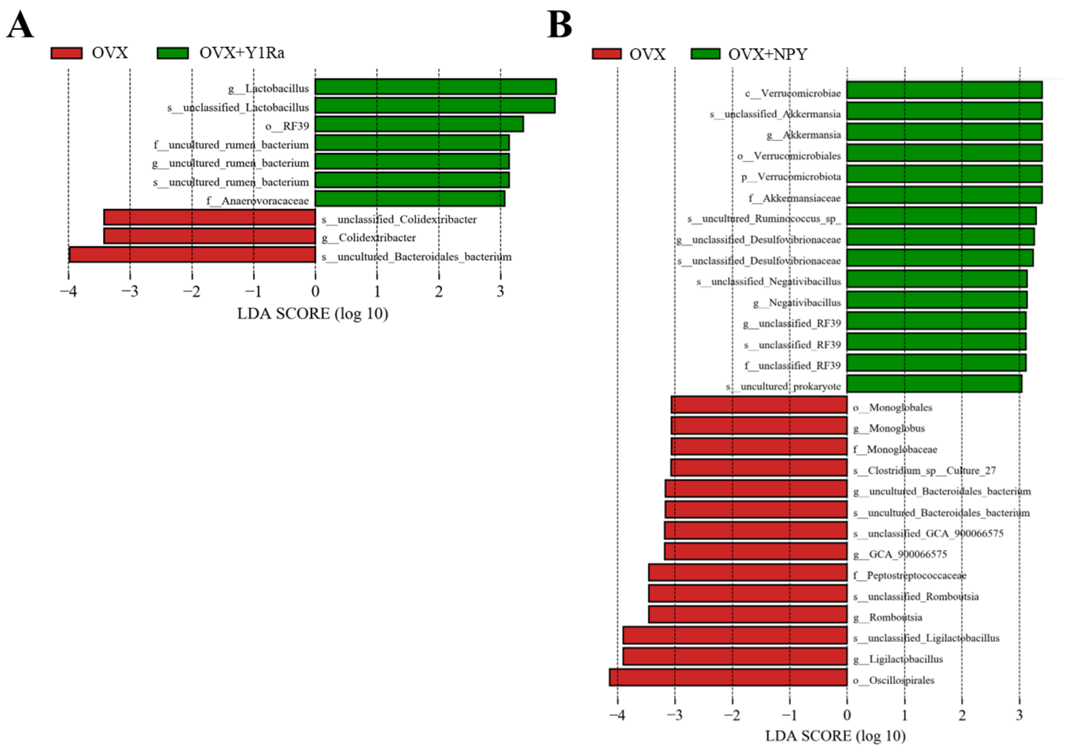

Supplementary Figure S4. The histograms of LDA value distribution of the LefSe analysis between OVX and OVX+Y1Ra rats, as well as between OVX and OVX+NPY rats, to find specific bacterial taxonomic markers**.** **(A)** g_Lactobacillus, s_unclassified_Lactobacillus, o_RF39, f_uncultured_rumen_bacterium, g_uncultured_rumen_bacterium, s_uncultured_rumen_bacterium and f_Anaerovoracaceae were found more enriched in the OVX+Y1Ra rats compared with the OVX rats. **(B)** c_Verrucomicrobiae, s_unclassified_Akkermansia, g_Akkermansia, o_Verrucomicrobiales, p_Verrucomivrobiota, f_Akkermansia, s_uncultured_Ruminococcus_sp, g_unclassified_Desulfovibrionaceae, s_unclassified_Desulfovibrionaceae, s_unclassified_Negativibacillus, g_Negativibacillus, g_ unclassified_RF39, s_ unclassified_RF39, f_ unclassified_RF39 and s_unclassified_prokaryote were found more enriched in the OVX+NPY rats compared with the OVX rats.

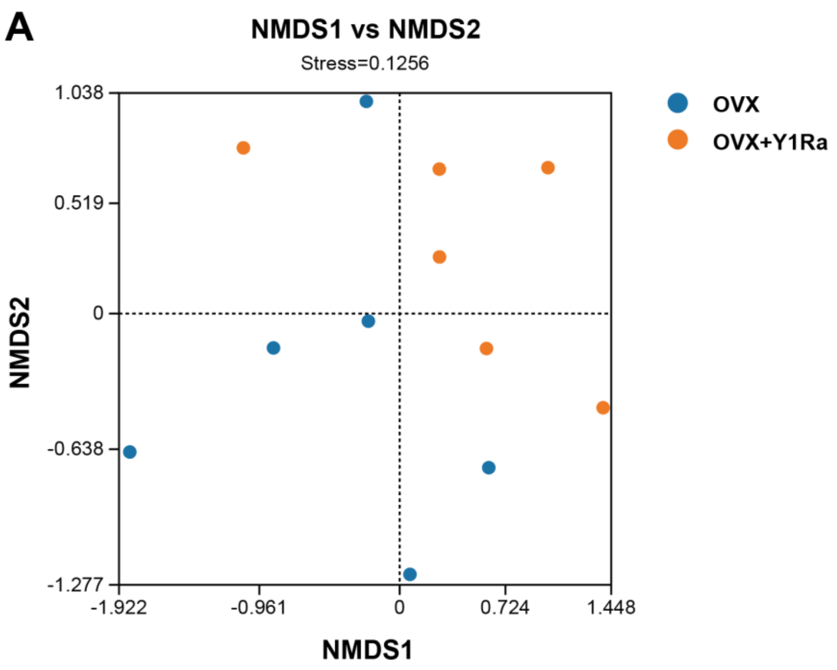

Supplementary Figure S5. (A) The NMDS analysis (NMDS1 vs NMDS2, stress=0.1256) based on the Binary_Jaccard showed an apparent separation in the structure and the community composition of GM between OVX rats and OVX+Y1Ra rats, illustrating that Y1Ra significantly changed the structures and the community compositions of GM in OVX rats.

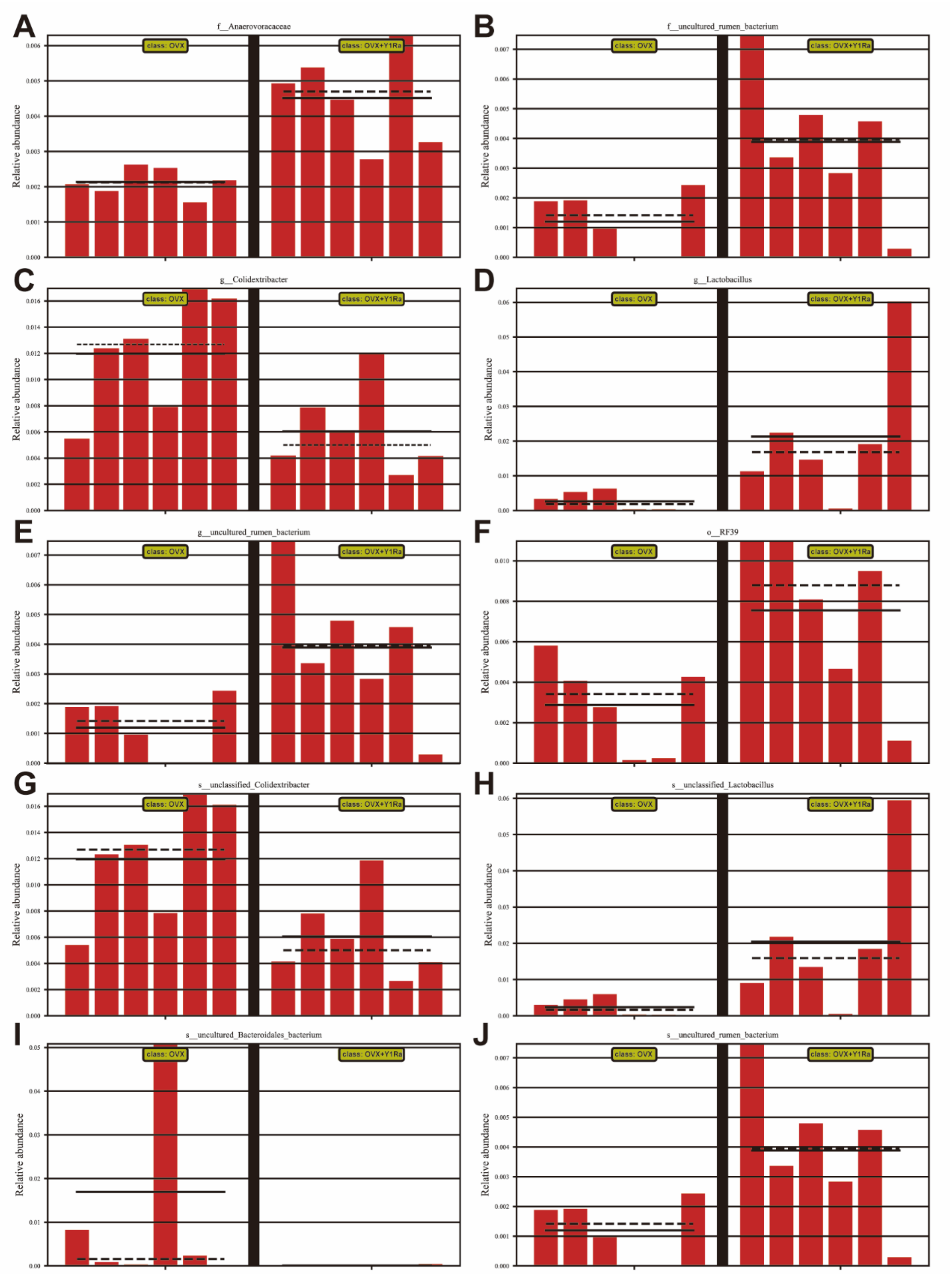

Supplementary Figure S6. The relative abundance of each sample within specific intestinal flora, which showed significant differences between the OVX and OVX+Y1Ra groups, including *g_Lactobacillus, s_unclassified_Lactobacillus, o_RF39, f_uncultured_rumen_bacterium, g_uncultured_rumen_bacterium, s_uncultured_rumen_bacterium and f_Anaerovoracaceae*.

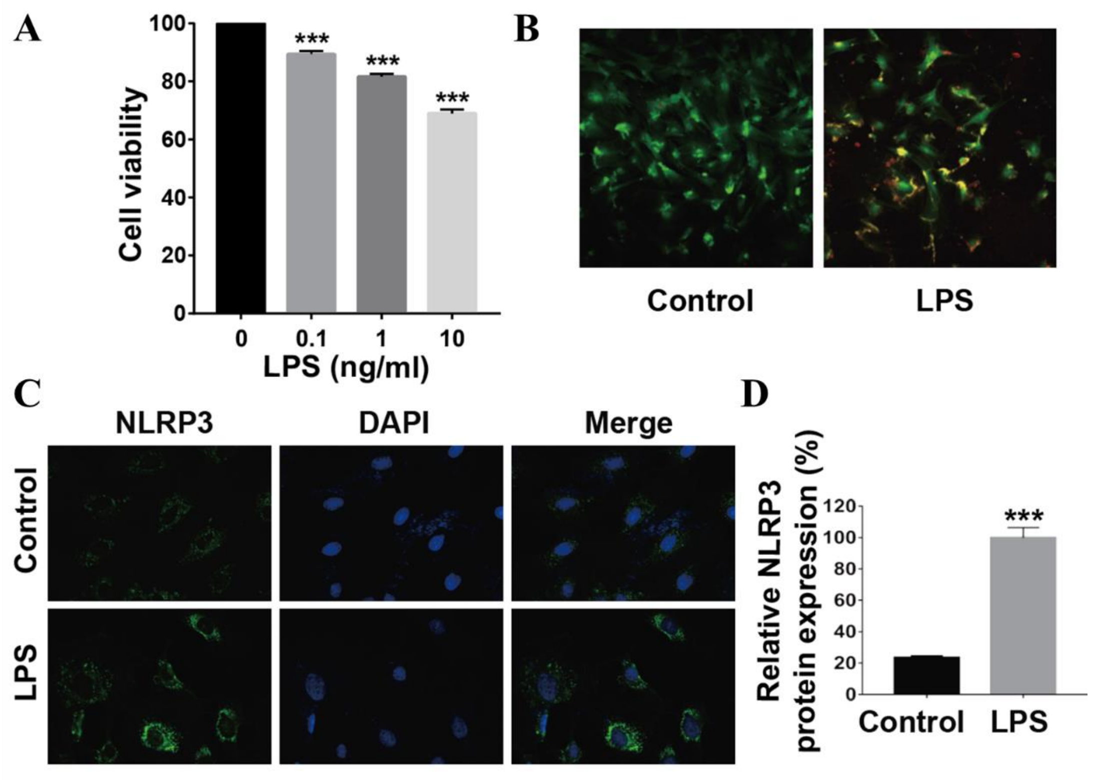

Supplementary Figure S7. LPS reduced viability and promoted the protein expression of NLRP3 of osteoblasts**.** **(A)** The CCK-8 test showed that cell viability of osteoblasts significantly decreased with treatment of LPS at the concentration from 0, 0.1, 1 to 10 ng/ml. **(B)** The Live/Dead staining showed that many dead cells were observed but still quite a few seeded cells stayed alive over the course of 3-day culture with 1 ng/ml LPS. **(C)** The immunofluorescence staining showed that NLRP3 was visibly higher in the LPS group (1 ng/ml) compared with the Control group. **(D)** The quantitative analysis showed that the relative protein expression of NLRP3 based on the fluorescence intensity.

Table S1. Comparison of the microstructural parameters of cancellous bone between SHAM, OVX, OVX+NPY and OVX+Y1Ra rats

**Parameters**	**SHAM**	**OVX**	**OVX+NPY**	**OVX+Y1Ra**
BV/TV (%)	62.500 ± 2.482	11.950 ± 1.947	4.611 ± 0.804	19.280 ± 2.253
BMD (g/cm^3)	0.471 ± 0.019	0.161 ± 0.014	0.092 ± 0.011	0.233 ± 0.017
Conn.D (mm^{-3})	144.300 ± 5.461	13.430 ± 2.017	11.150 ± 3.172	30.370 ± 5.248
Tb.N (mm^{-1})	3.475 ± 0.122	1.352 ± 0.105	0.996 ± 0.123	1.942 ± 0.148
Tb.Sp (μm)	148.600 ± 15.680	741.200 ± 50.970	966.300 ± 76.750	503.700 ± 54.880
Tb.Th (μm)	143.300 ± 5.265	125.500 ± 7.594	118.100 ± 9.771	122.900 ± 6.698

Table S2. The α-diversity original data comparison among four groups

Sample ID	Shannon index	Simpson index	ACE index	Chao1 index
SHAM 1	6.1396	0.9633	484.4827	488.6667
SHAM 2	6.3577	0.9719	500.1325	501
SHAM 3	6.926	0.9843	492.372	511.0625
SHAM 4	6.3158	0.973	508.0435	511.1818
SHAM 5	6.198	0.9651	499.4289	504.129
SHAM 6	5.4431	0.946	424.626	443.4545
OVX 1	6.7165	0.9803	526.595	525.5
OVX 2	6.4419	0.9695	513.8193	519.6667
OVX 3	6.7364	0.9798	525.696	532.3704
OVX 4	6.1359	0.9702	464.5744	479.4167
OVX 5	6.4461	0.9729	456.4895	491.0667
OVX 6	6.673	0.9772	515.6216	533
OVX+NPY 1	6.3767	0.9653	557.0425	581.3
OVX+NPY 2	6.218	0.9491	586.6616	590.1176
OVX+NPY 3	6.6877	0.9719	592.8595	592.0303
OVX+NPY 4	7.1621	0.9822	593.4426	598.2174
OVX+NPY 5	6.408	0.9652	565.5811	571.2188
OVX+NPY 6	6.015	0.9595	549.5051	555.4167
OVX+Y1Ra 1	6.3088	0.9656	496.9192	519.5882
OVX+Y1Ra 2	6.365	0.9667	500.4183	499.7308
OVX+Y1Ra 3	6.8557	0.9818	519.2326	523.913
OVX+Y1Ra 4	6.3543	0.9624	547.3849	559.0333
OVX+Y1Ra 5	6.6249	0.9775	519.4849	525.3333
OVX+Y1Ra 6	6.0816	0.968	474.1398	473.8235

Table S3. The original data for β-diversity comparison among four groups

Sample ID	PC1	PC2	PC3	PC4	PC5
SHAM 1	-0.1725	-0.1384	-0.2009	-0.0902	0.0104
SHAM 2	-0.0702	-0.1333	0.0417	0.0243	0.0537
SHAM 3	-0.0734	-0.089	0.0571	0.053	0.058
SHAM 4	-0.0561	-0.1253	0.065	0.0431	0.0247
SHAM 5	-0.0866	-0.0906	-0.0406	0.0208	-.0353
SHAM 6	-0.039	-0.1164	0.0701	0.0658	-.0299
OVX 1	-0.0169	0.0839	0.0384	-0.0164	0.0118
OVX 2	-0.0459	0.0393	0.0701	-0.0513	0.0313
OVX 3	-0.0301	0.067	0.0297	-0.0487	0.0333
OVX 4	-0.1146	0.0995	-0.0306	0.0357	0.023
OVX 5	-0.0515	0.0731	0.0375	0.0249	0.0164
OVX 6	-0.0491	0.1344	-0.0916	0.1483	-.0216
OVX+NPY1	0.2096	-0.036	-0.0208	-0.0069	0.0313
OVX+NPY2	0.2271	0.0042	-0.0482	0.0212	0.0304
OVX+NPY3	0.21	-0.0154	-0.0332	-0.0182	0.0419
OVX+NPY4	0.207	-0.0092	-0.0329	0.001	0.0447
OVX+NPY5	0.0873	-0.0117	0.0051	-0.0003	-0.1007
OVX+NPY6	0.0766	-0.0555	-0.0081	0.0036	-0.1387
OVX+Y1Ra 1	-0.0302	0.0299	0.0669	-0.0704	-0.0018
OVX+Y1Ra 2	-0.0353	0.0369	0.0141	-0.0374	-0.043
OVX+Y1Ra 3	0.0033	0.0244	0.0403	-0.0217	-0.0312
OVX+Y1Ra 4	-0.022	0.061	0.0487	-0.0501	-0.0292
OVX+Y1Ra 5	-0.0403	0.062	-0.0016	-0.0369	-0.0082
OVX+Y1Ra 6	-0.0874	0.1051	-0.0763	0.0067	0.0286

Table S4. The original data for composition and structure of GM at the phylum level among four groups

phylum	SHAM	OVX	OVX+NPY	OVX+Y1Ra
Firmicutes	0.58621	0.665924	0.663504	0.649175
Bacteroidota	0.404526	0.325002	0.31375	0.336753
Desulfobacterota	0.004064	0.003938	0.008042	0.003329
Proteobacteria	0.002239	0.001722	0.004153	0.005179
Actinobacteriota	0.001433	0.001402	0.001329	0.002333
Cyanobacteria	0.001489	0.00146	0.001188	0.001272
Verrucomicrobiota	0	1.12E-05	0.004966	4.45E-06
Patescibacteria	6.70E-06	0.000505	0.002387	0.001948
Elusimicrobiota	0	2.24E-06	0.000382	0
Deferribacterota	3.35E-05	3.35E-05	0.0003	6.67E-06

Table S5. The original data for composition and structure of GM at the class level among four groups

class	SHAM	OVX	OVX+NPY	OVX+Y1Ra
Clostridia	0.559662	0.623915	0.626989	0.56649
Bacteroidia	0.404526	0.325002	0.31375	0.336753
Bacilli	0.026547	0.042004	0.036514	0.082685
Desulfovibrionia	0.004064	0.003938	0.008042	0.003329
Gammaproteobacteria	0.002239	0.001722	0.004153	0.005179
Vampirivibrionia	0.001489	0.00146	0.001188	0.001272
Verrucomicrobiae	0	1.12E-05	0.004966	4.45E-06
Saccharimonadia	6.70E-06	0.000505	0.002387	0.001948
Coriobacteriia	0.000382	0.000984	0.000479	0.001663
Actinobacteria	0.001051	0.000418	0.00085	0.000669
Elusimicrobia	0	2.24E-06	0.000382	0
Deferribacteres	3.35E-05	3.35E-05	0.0003	6.67E-06
Negativicutes	0	4.47E-06	0	0

Table S6. The original data for composition and structure of GM at the order level among four groups

order	SHAM	OVX	OVX+NPY	OVX+Y1Ra
Bacteroidales	0.404488	0.324772	0.313296	0.335991
Lachnospirales	0.304621	0.373491	0.405636	0.28447
Oscillospirales	0.191829	0.19818	0.166555	0.203584
Clostridia_UCG_014	0.030487	0.019743	0.031589	0.038458
Lactobacillales	0.006441	0.020626	0.018203	0.03903
Erysipelotrichales	0.014089	0.00979	0.004462	0.022381
Peptostreptococcales_Tissierellales	0.012163	0.011782	0.006595	0.014309
Peptococcales	0.006026	0.007554	0.004532	0.006642
unclassified_Bacilli	0.001839	0.004678	0.006099	0.012099
Christensenellales	0.006921	0.003571	0.006913	0.005977
RF39	0.003727	0.002885	0.006624	0.007587
Desulfovibrionales	0.004064	0.003938	0.008042	0.003329
Monoglobales	0.004682	0.004727	0.002567	0.006364
Enterobacterales	0.001024	0.001398	0.002583	0.00386
unclassified_Clostridia	0.001044	0.001485	0.000704	0.003983
Clostridia_vadinBB60_group	0.001558	0.002281	0.001656	0.001543
Acholeplasmatales	0	0.003996	0.001056	0.001436
Gastranaerophilales	0.001489	0.00146	0.001188	0.001272
Verrucomicrobiales	0	1.12E-05	0.004966	4.45E-06
Saccharimonadales	6.70E-06	0.000505	0.002387	0.001948
Others	0.003499	0.003126	0.004348	0.00573

Table S7. The original data for LEfSe features of four groups

Biomarkers	Abundance	Group	LDA	P value
g__Bacteroides	4.538656	SHAM	4.170785	0.003493
f__Bacteroidaceae	4.538656	SHAM	4.170785	0.003493
s__unclassified_Ruminococcus	4.575531	SHAM	4.143199	0.019674
g__Ruminococcus	4.593632	SHAM	4.108601	0.03376
s__Phocaeicola_barnesiae	4.008748	SHAM	3.667107	0.008081
s__unclassified_Bacteroides	4.022155	SHAM	3.635499	0.000705
s__unclassified_Parabacteroides	4.086302	SHAM	3.578894	0.046706
f__Peptostreptococcaceae	3.994216	SHAM	3.506629	0.03729
s__unclassified_Romboutsia	3.994216	SHAM	3.506629	0.03729
g__Romboutsia	3.994216	SHAM	3.506629	0.03729
s__Bacteroides_coprocola	3.686887	SHAM	3.402664	0.001306
s__Bacteroides_sp__Marseille_P3208T	3.424567	SHAM	3.208663	0.049503
s__uncultured_Clostridium_sp_	3.41988	SHAM	3.117562	0.0288
s__unclassified_UCG_008	2.803783	SHAM	3.105764	0.020296
s__unclassified_Ligilactobacillus	4.252294	OVX	3.919844	0.003493
g__Ligilactobacillus	4.252294	OVX	3.919844	0.003493
s__uncultured_Bacteroidales_bacterium	4.22896	OVX	3.888076	0.00609
s__unclassified_Anaeroplasma	3.602263	OVX	3.278887	0.001258
f__Acholeplasmataceae	3.602263	OVX	3.278887	0.001258
g__Anaeroplasma	3.602263	OVX	3.278887	0.001258
o__Acholeplasmatales	3.602263	OVX	3.278887	0.001258
s__uncultured_Bacteroidales_bacterium	3.515752	OVX	3.225774	0.000395
g__uncultured_Bacteroidales_bacterium	3.515752	OVX	3.225738	0.000395
s__Clostridium_sp__Culture_27	3.495667	OVX	3.218065	0.030782
g__Prevotella_9	4.015685	OVX+NPY	3.728574	0.010518
s__unclassified_Prevotella_9	4.015685	OVX+NPY	3.728574	0.010518
s__uncultured_prokaryote	3.97653	OVX+NPY	3.631925	0.001452
g__Akkermansia	3.696234	OVX+NPY	3.411446	0.000783
s__unclassified_Akkermansia	3.696234	OVX+NPY	3.411446	0.000783
f__Akkermansiaceae	3.696234	OVX+NPY	3.411446	0.000783
o__Verrucomicrobiales	3.696234	OVX+NPY	3.411446	0.000783
p__Verrucomicrobiota	3.696234	OVX+NPY	3.411446	0.000783
c__Verrucomicrobiae	3.696234	OVX+NPY	3.411446	0.000783
g__unclassified_Desulfovibrionaceae	3.574453	OVX+NPY	3.335068	0.001399
s__unclassified_Desulfovibrionaceae	3.552065	OVX+NPY	3.2922	0.001399
s__uncultured_Ruminococcus_sp_	3.552379	OVX+NPY	3.242695	0.001183
s__unclassified_Negativibacillus	3.450271	OVX+NPY	3.138604	0.002979
g__Negativibacillus	3.450271	OVX+NPY	3.138604	0.002979
g__Candidatus_Saccharimonas	3.378874	OVX+NPY	3.092736	0.000896
f__Saccharimonadaceae	3.378874	OVX+NPY	3.092648	0.000896
o__Saccharimonadales	3.378874	OVX+NPY	3.0926	0.000896

s__unclassified_Candidatus_Saccharimonas	3.378874	OVX+NPY	3.092579	0.000896
p__Patescibacteria.c__Saccharimonadia	3.378874	OVX+NPY	3.092575	0.000896
p__Patescibacteria	3.378874	OVX+NPY	3.092534	0.000896
c__Bacilli	4.918137	OVX+Y1Ra	4.482211	0.029202
o__Lactobacillales	4.591686	OVX+Y1Ra	4.242757	0.048413
g__Lactobacillus	4.329061	OVX+Y1Ra	4.025455	0.017307
s__unclassified_Lactobacillus	4.309936	OVX+Y1Ra	4.011379	0.02016
s__unclassified_Incertae_Sedis	4.247518	OVX+Y1Ra	3.837626	0.020532
g__Incertae_Sedis	4.259413	OVX+Y1Ra	3.833612	0.030836
f__Anaerovoracaceae	3.654793	OVX+Y1Ra	3.08269	0.00859

Table S8. Comparison of the microstructural parameters of cancellous bone between PBS, trans-SHAM, trans-OVX, trans-OVX+NPY and trans-OVX+Y1Ra rats

**Parameters**	**PBS**	**trans-SHAM**	**trans-OVX**	**trans-OVX+NPY**	**trans-OVX+Y1Ra**
BV/TV (%)	41.530 ± 1.909	48.820 ± 1.866	40.960 ± 1.853	34.040 ± 2.390	44.570 ± 1.575
BMD (g/cm^3)	0.344 ± 0.013	0.412 ± 0.013	0.324 ± 0.011	0.285 ± 0.017	0.394 ± 0.013
Conn.D (mm^{-3})	127.300 ± 3.140	139.900 ± 7.260	125.500 ±5.418	115.700 ± 7.221	129.600 ± 7.679
Tb.N (mm^{-1})	2.906 ± 0.119	3.295 ± 0.144	2.958 ± 0.155	2.737 ± 0.142	3.156 ± 0.159
Tb.Sp (μm)	187.900 ± 7.257	160.100 ± 9.191	197.200 ± 6.849	222.500 ± 12.900	174.400 ± 6.522
Tb.Th (μm)	133.100 ± 6.699	132.200 ± 6.987	131.400 ± 8.252	120.900 ± 6.122	130.500 ± 6.072

Table S9. Colonic histopathological scores

Score	Inflammation	Crypt Damage	Ulceration	Edema
0	No infiltrate	None	None	None
1	Occasional cell limited to submucosa	Some crypt damage, spaces between crypts	Small, focal ulcers	Present
2	Significant presence of inflammatory cells in submucosa, limited to focal areas	Larger spaces between crypts, loss of goblet cells, some shortening of crypts	Frequent small ulcers	
3	Infiltrate present in both submucosa and lamina propria, limited to focal areas	Large areas without crypts, surrounded by normal crypts	Large areas lacking surface epithelium	
4	Large amount of infiltrate in submucosa, lamina propria and surrounding blood vessels, covering large areas of mucosa	No crypts		
5	Transmural inflammation (mucosa to muscularis*)*			
